# Effectiveness of orofacial myofunctional intervention to mitigate facial aging signs: a clinical trial

**DOI:** 10.1590/2317-1782/20242023016en

**Published:** 2024-08-19

**Authors:** Yasmin Salles Frazão, Silvia Bertacci Manzi, Lilian Krakauer, Giédre Berretin-Felix

**Affiliations:** 1 Programa de Pós-graduação em Fonoaudiologia, Faculdade de Odontologia de Bauru, Universidade de São Paulo – USP - Bauru (SP), Brasil.; 2 Conselho Federal de Fonoaudiologia – CFFa - São Paulo (SP) Brasil.; 3 Pontifícia Universidade Católica de São Paulo – PUCSP - São Paulo (SP), Brasil.; 4 Departamento de Fonoaudiologia, Faculdade de Odontologia de Bauru, Universidade de São Paulo – USP - Bauru (SP), Brasil.

**Keywords:** Esthetics, Speech, Language and Hearing, Sciences, Myofunctional Therapy, Electromyography, Rejuvenation, Aging

## Abstract

**Purpose:**

Propose and verify the efficiency of myofunctional intervention program to attenuate facial aging signs and balance the orofacial functions.

**Methods:**

Thirty women, aged 50 to 60 years, randomly divided into: therapy group (TG) submitted to Orofacial Myofunctional Therapy and electromyographic biofeedback group (EBG), submitted to the same program associated with electromyographic biofeedback for chewing, swallowing, and smiling functions training. Aesthetic and oromyofunctional aspects were assessed from photographs, videos, MBGR Protocol and scales for assessing facial aging signs, described in the literature. 50-minute sessions were held weekly for nine weeks and monthly for six months after washout period. Three assessments, identical to the initial one, were performed in the tenth week, eighth week after washout and conclusion of the research. The participants responded to the Satisfaction Questionnaire on the tenth week.

**Results:**

The statistical analysis using the ANOVA, Tukey and Mann Whitney tests, for inter and intragroup comparison, showed that: intervention promoted attenuation of facial aging signs mainly in TG group, balance of chewing and swallowing functions in both groups; there was an impact of electromyographic biofeedback on the degree of participants’ satisfaction, greater for EBG; interruption of the program for eight weeks resulted in aesthetic losses, mainly in TG, yet not functional losses, in both groups; the six monthly sessions had a limited impact on overcoming the esthetic losses that occurred after washout.

**Conclusion:**

The proposed program resulted in attenuation of aging signs, mainly in the TG group and improvement in orofacial functions, in both groups.

## INTRODUCTION

Facial aging results from several factors inherent to the natural aging process and also from lifestyle-related factors, which can increase the appearance of facial wrinkles and *sulci*, such as exposure to the sun without sunscreen^(1,2)^, harmful habits such as smoking, an unbalanced diet, and a sedentary lifestyle^(1,3)^. Exaggerated contraction of muscles involved in orofacial functions is another factor frequently reported by doctors and speech pathologists^(4-7)^ and can result in static and/or dynamic wrinkles, depending on the intensity, frequency, and duration of these contractions, as well as the individual dentoskeletal characteristics^(6,8)^.

Several professionals dedicate themselves to research and work in facial aesthetics using clinical resources, more or less invasive, to reduce facial aging. Various surgical techniques and procedures, such as facial fillers, botulinum toxin and laser are some of the resources proposed by dermatologists and plastic surgeons^(2,9)^. The orofacial myofunctional therapy (OMT) proposed by speech pathologists is a non-invasive approach that meets the demand of clients who embrace the natural aging process^(5-7)^.

It was found that there is a wide variety of clinical resources used by speech pathologists in facial aesthetics interventions. For some, muscle contraction exercises, performed after muscle relaxation maneuvers, would be essential to reduce facial wrinkles; on the other hand, there are those who condemned the use of muscle contraction exercises; others proposed the combination of these exercises with adequation of orofacial functions^(10)^ and there are also some who used thermotherapy, cryotherapy^(11)^, and neuromuscular electrical stimulation^(12)^. Although surface electromyography (SE) is considered a valuable instrument for both the diagnosis and rehabilitation of individuals with myofunctional orofacial alterations resulting from different etiologies^(13)^, in only one study was electromyographic biofeedback (EBF) used as resource in speech therapy intervention for facial aesthetics^(14)^. Researchers from this and other studies^(15,16)^ agree that this technique represents a promising adjunct modality in the therapeutic process.

In most of the consulted publications^(5-7,11,12)^, the authors described significant aesthetic changes and stated that the various exercises employed mitigated aging signs. However, the evidence described in these studies can be questioned when analyzing their inaccurate methodology, as Valente points out in a systematic review^(10)^. Most of the studies had a small number of subjects, evaluated by the researchers themselves, a fact that would reduce the degree of reliability of these results^(10)^. Such findings demonstrate the need for research with greater methodological rigor and quantitative measurement of results. The present study is the first clinical trial in which a therapeutic program aimed at reducing facial aging signs was tested in its entirety.

Therefore, the objective of this study was to propose and verify the efficiency of an orofacial myofunctional intervention program to mitigate the facial aging signs and balance orofacial functions. To this end, it was verified whether there would be aesthetic and orofacial myofunctional regression with the interruption of the therapeutic program (washout period); whether the results obtained by the electromyographic biofeedback group (EBG) would be better than those of the therapy group (TG); whether the degree of satisfaction of the volunteers, in relation to the therapeutic program, would be compatible with the results measured in the Orofacial Myofunctional Evaluation Protocol MBGR and in the Facial Aging Signs Analysis (FASA); whether carrying out six sessions, once a month, for six months, would be sufficient to reverse the aesthetic and functional regressions after the washout period.

## METHOD

This was a randomized controlled clinical trial, approved by the Ethics Committee, opinion number 2.235.918 – CAAE: 71680017.0.0000.5417. All participants were duly informed about the objectives and procedures carried out in the research and signed the Free and Informed Consent Form.

The female research participants were between 50 and 60 years old (53.73 ± 2.79). The exclusion criteria adopted were: carrying out invasive facial procedures (plastic surgery, facial fillers, botulinum toxin application, laser application) and non-invasive facial procedures (drainage; massages; medications; new creams, other than those used habitually) in the year prior to the start of treatment and during participation in the research, history of dentofacial skeletal deformity, temporomandibular disorder, presence of snoring, partial edentulism (multiple tooth loss), intolerance to foods used in the assessment and therapy, unavailability to comply with the research schedule for 42 weeks.

Through a sample calculation carried out based on a pilot study, the number of 30 volunteers was determined, who were randomly distributed into two groups using a Microsoft Office Excel spreadsheet: therapy group (TG), submitted to the orofacial myofunctional therapy program determined by the project; electromyographic biofeedback group (EBG), submitted to the same therapeutic program with electromyographic biofeedback used in parallel for chewing, swallowing and smiling training.

The participants underwent an initial assessment, which included photographic and video documentation of some of the tests included in the Orofacial Myofunctional Assessment Protocol MBGR^(17)^. To carry out this documentation, the participants were dressed in a light blouse without a collar, without makeup, without earrings. The standard for photographic and video documentation was proposed by Frazão and Manzi (2019)^(18)^. Three assessments, identical to the initial one, were carried out respectively in the 10th session, in the eighth week after washout, and at the conclusion of the research at the end of the six months following the washout. The participants responded to the Satisfaction Questionnaire in the tenth week on printed paper without identifying themselves before the photographic and video documentation, and in the absence of the researcher in the room. The completed questionnaires were sealed, placed in an equally sealed plastic envelope in the presence of the participant and, later, delivered to the evaluating speech pathologists.

After the initial assessment, participants in both groups underwent a therapeutic intervention lasting nine weeks (one session weekly), during which they learned isotonic and isometric exercises (buccinator muscles, palpebral portion of the *orbicularis oculi* muscle and suprahyoid muscles), and adequate patterns of chewing, swallowing and facial expression control during communication/speech. After this initial stage, there was a period of eight weeks in which the program was interrupted (washout). In the six months following the washout, the sessions were resumed and carried out once a month.

**Figure 1:**
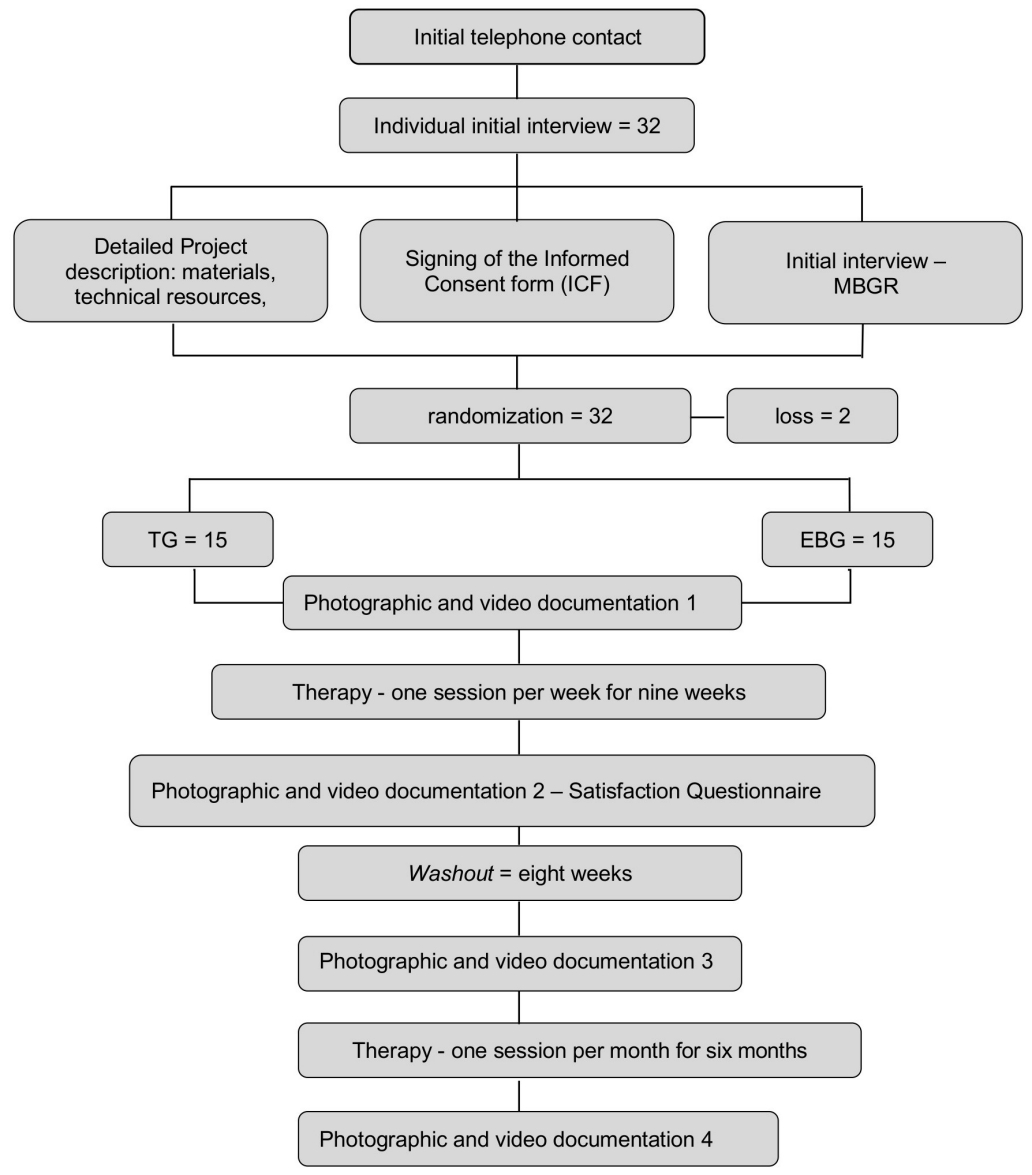
Flowchart of the Research Steps

The photographic and video documentation of the thirty participants in the four assessments carried out (T1, T2, T3 and T4, totaling one hundred and twenty assessments) were randomly distributed and sent to the two speech pathologists, specialists in Orofacial Motricity, with thirty-four and thirty-eight years of clinical experience, previously calibrated. The calibration was completed when the evaluators reached a minimum reliability percentage of 90%. The scores from the MBGR Protocol and the Facial Aging Signs Analysis (FASA), based on photonumeric scales validated in the literature^(19-22)^, were checked by the evaluators, who also compiled the scores recorded in the Satisfaction Questionnaire. The evaluators received photos in the front, 45-degree and right and left profile angles to check static wrinkles, filming of orofacial functions (speech, chewing and swallowing) and crop video images (print screen) to evaluate dynamic wrinkles, which recorded the contraction of the muscles: frontal portion of the *occipitofrontalis* muscle, corrugator *supercilii*, *orbicularis oculi* and *orbicularis oris*. The evaluators carried out a blind evaluation of this documentation and decided, by consensus, on the divergent answers, reaching an agreement of 92% for the MBGR Protocol and 98% for the FASA. Figure 1 shows the flowchart of the research steps.

In this study, which began in 2017, some tests were selected from the Orofacial Myofunctional Clinical Examination, 2014 version of the MBGR Protocol: orofacial functions (habitual chewing, habitual swallowing of solid food), directed swallowing (water), speech; general aspects (swallowing saliva during speech tests). In this Protocol, a higher score is assigned to pronounced orofacial myofunctional alterations and a lower score to minor alterations. For data analysis, the total sum of the chewing, swallowing and speech function scores assessed using the MBGR Protocol was considered.

A photonumeric scale was created from the selection of some images from the validated scales proposed by Flynn et al.^(19)^, Narins et al.^(20)^, Carruthers et al.^(21)^, Jones et al.^(22)^ to assess wrinkles and aging signs in the upper, middle and lower facial thirds in the four assessments carried out. In accordance with what was recommended by the authors of these scales, values were assigned to static and dynamic frontal, glabellar and periorbital wrinkles^(19)^; perioral wrinkles, nasolabial and mentolabial *sulci*, ptosis in the mandibular (frontal) and submental contour (profile)^(20)^; wrinkles on the cheeks^(21)^; and cervical wrinkles^(22)^. The value zero was assigned to the absence of wrinkles, *sulci* and ptosis and the value 4 to the pronounced presence of these aging signs. The maximum (56) and minimum (zero) values attributed, respectively, to the greater or lesser presence of facial aging signs were registered in a table - Facial Aging Signs Analysis (FASA), being the total sum of the scores of the aesthetic aspects assessed, the numerical value considered for the statistical analysis of the data.

The Satisfaction Questionnaire, with a maximum score of 90, was responded to by the participants after the initial nine sessions of the proposed program, before viewing their photographic and video documentation, registered in the T1 and T2 assessments. The total sum of the values referring to the participants' responses was considered for the statistical analysis of the data.

## THERAPEUTIC PROGRAM: OROFACIAL MYOFUNCTIONAL THERAPY TO REDUCE FACIAL AGING SIGNS (OMTFAS)

The therapeutic program consisted of two stages: in the first, nine sessions were held weekly; in the second, six sessions were held monthly, starting after the washout period. Participants in both groups underwent the same therapeutic program. The collection of signs and functional training of chewing, swallowing and smiling for the EBG participants will be described in the section – **Training with Electromyographic Biofeedback - EBG** **group**.

The therapeutic objectives were advanced during the sessions held in the two stages of the therapeutic program, through appropriate strategies. The exercises were presented gradually at each session, and, at the end of the sessions, the participants received updated printed guidelines that should have been carried out daily in their homes and handed the printed sheet containing the previous guidelines to the researcher. Although there was no control over the performance of the instructions at home, the researcher was able to observe changes in the motor patterns trained each week. The effective performance of the exercises prescribed to do at home was verified by the improvement in the performance of each exercise in each session. All procedures used in the therapeutic program were described in detail to ensure that this study can be replicated and utilized by other researchers, and can be accessed on the website of the Biblioteca Digital de Teses e Dissertações da USP^(23)^

**Figure 2:**
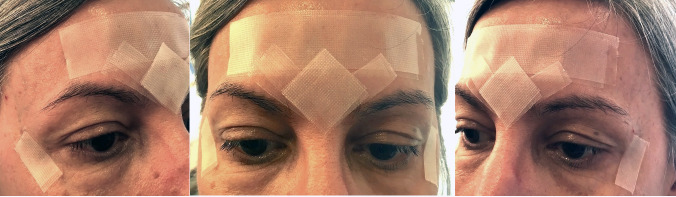
Application of the non-elastic bandage - Transpore™ - across the forehead and glabella regions (Transpore™ with a 25 mm width), V-shaped glabella, and the external canthi of the eyes (Transpore™ with a 12 mm width). Application of a double layer of tape (one atop the other) is executed in all specified areas (forehead, glabella, and external eye corners)

The following objectives and strategies, which will be briefly described, were implemented in therapy: manual stretching of the right and left temporalis and masseter muscles with the aim of reducing their excessive contraction; maintenance, during the day, of two centimeters of tourniquet, five millimeters in diameter in the oral vestibule (intersection between the mentalis muscle and lower lip), with the aim of reducing contraction of the perioral region. Use of the Transpore^TM^ inelastic bandage (Figure 2)

during nocturnal sleep on the occipitofrontal, *corrugator supercilii* and *orbicularis oculi* muscles (outer corner of the eyes) to reduce contraction of the muscles in the upper and middle third of the face. The participants were trained in therapy to place the Transpore^TM^ properly and also recorded, on their cell phones, a photograph of the inelastic bandage properly positioned on their faces. Carrying out isometric and isotonic exercises to condition the muscles in the cheek region, suprahyoid muscles, lingual muscles and reduce nasolabial sulcus, cheek ptosis and submental ptosis using the Facial Plus and Lingual exercisers – Pró-Fono^®^. Carrying out isotonic and resistance exercises to condition the palpebral portion of the *orbicularis oculi* muscle in order to keep eyes wider and reduce the contraction of the frontalis, *corrugator supercilii* and *orbicularis oculi* muscles during communication. Carrying out the cork exercise (speak with a wine cork between the teeth), keeping the mouth open by the diameter of the cork, as a technique to reduce excessive contraction of the perioral muscles when articulating speech. Carrying out functional training to adapt chewing and swallowing patterns with liquid, solid food and Halls® candy, a soft smile (“social smile”), without lip sealing and control of facial expressions during communication.

In the tenth week, when the second assessment was carried out, the participants returned the printed sheet with the guidelines, as well as all the materials used during the nine sessions (facial and tongue exercisers, tourniquet, wine cork, Transpore^TM^ inelastic bandage and Halls^®^ candy); the collection of all materials and the printed sheet with the guidelines for carrying out the exercises at home was the procedure adopted to ensure that the exercises were not performed during the washout period. The materials were stored in individual plastic bags (except the Halls^®^ candy, which was discarded; a new box of candy was given to the participants after the washout), identified with the participant's name, and later returned in the second stage of the program.

The second stage of the therapeutic program began after the eight-week washout period, when the participants were assessed (third assessment) and began the sequence of six monthly sessions. On this occasion, the exercises and guidelines that should be practiced by the participants daily in their homes were reviewed; the guidelines delivered on a printed sheet in the first session after the washout were the same as those received in the ninth session. At the end of the six months, the participants received the following final instructions, on a printed sheet, with the recommendation to carry them out daily, for life: use of the Transpore^TM^ inelastic bandage, execution -- isotonic and isometric exercises for the eyes and cheek region (facial exerciser), tongue and suprahyoid muscles (lingual exerciser); maintenance of functional control of chewing, swallowing and speech, but without repeating the initial functional training. The participants were instructed to perform the pressure maneuver at the intersection between the mentalis muscle and lower lip, replacing the use of a tourniquet and stretching of the masseter and temporal muscles, when necessary, i.e., when they notice exaggerated muscle contraction in these facial regions.

## TRAINING WITH ELECTROMYOGRAPHIC BIOFEEDBACK - EBG GROUP

The collection of signals and functional training of chewing, swallowing and smiling of EBG participants was carried out with the Biotrainer software on Miotec’s New Miotool Face device, which has eight channels connected to differential active sensors, with 16-bit resolution, 2kHz sampling frequency, 20Hz low-pass filter, 500Hz high-pass filter, 60Hz notch, with clamp connection and one reference (ground). To capture the electrical signal, with the same device, double passive differential electrodes, Double Trace LH-ED4020, were used; dimensions: 44 mm long, 21 mm wide, 20 mm center to center, placed over muscles involved in chewing, swallowing and smiling (right and left masseter; zygomaticus major, minor, risorius; *orbicularis oris* – upper lip; suprahyoid; lower orbital portion of the *orbicularis oculi* muscle). The reference electrode (ground) was positioned over the styloid process of the ulna of participants’ right arm. The areas where the electrodes were positioned were previously cleaned with gauze and 70% isopropyl alcohol in all sessions in which training with electromyographic biofeedback was carried out.

The maximum voluntary contraction (MVC) of the selected muscles was measured and the value of 50% of the MVC was the parameter established for increasing or decreasing muscle contraction during training, that is, reduce contraction of the masseter, *orbicularis oris*, zygomaticus major and minor, and *orbicularis oculi* muscles; increase contraction of the suprahyoid muscles. The percentage of muscle contraction intensity can be established in the software, in the Collection Configuration window, Protocols tab, Activities tab, New Activity window, Intensity (%). Participants were instructed to increase muscle contraction, exceeding the target line, or reduce muscle contraction, remaining below the target line.

For the electromyographic biofeedback training, the electrodes were placed in a sequence to favor the participants' progressive control over the movements of the different muscle groups when performing the trained orofacial functions.

Thus, for the alternating unilateral chewing training, started in the second session, the electrodes were positioned parallel to the direction of the muscle fiber, over the right and left masseter muscles, and the participants were instructed to chew a full portion on one side and another full portion on the other side; in the third and fourth sessions, the electrodes were positioned over the right and left masseter muscles and *orbicularis oris* muscle (upper lip), with a requirement for lip contraction below an average of 50% of the MVC^(24)^ to reduce the possibility of formation of dynamic and static wrinkles in the perioral region; finally, for the alternating bilateral chewing training, in which the same portion is chewed on one side and then on the other side, carried out from the fifth to the ninth session, the electrodes were positioned over the right and left masseter muscles, *orbicularis oris* muscle (upper lip) and region of suprahyoid muscles, since in these sessions the chewing training was associated with the swallowing of crushed raisins and participants were instructed to contract the suprahyoid muscles above an average of 50% of the MVC when swallowing.

For the swallowing training, started in the third session with the swallowing of pasty food (Greek yogurt), the electrodes were positioned in the region of the suprahyoid muscles; in the fourth and fifth sessions (water swallowing), the electrodes were positioned in the region of the suprahyoid muscles, *orbicularis oris* muscle (upper lip); finally, for the swallowing training of crushed raisins, from the fifth to ninth session, the electrodes were positioned in the region of the suprahyoid, *orbicularis oris* (upper lip) and right and left masseter muscles. During swallowing, the participants were instructed to contract the suprahyoid muscles above an average of 50% of the MVC, with a consequent increase in the contraction of the tongue muscles and greater ejection force^(25)^, and to maintain a reduced contraction of the masseter muscles and upper lip, just enough to stabilize the jaw and keep the lips closed without tension.

In the smile training, started in the fifth session, the electrodes were initially positioned in the region of the left and right risorius muscles, and participants were instructed to give a soft smile, without lip sealing, while thinking about something happy; subsequently, from the sixth to the ninth session, the electrodes were positioned in the region of the left and right risorius muscles and in the lower orbital portion of the right and left *orbicularis oculi* muscle, and participants were instructed to keep the contraction of the *orbicularis oculi* below an average of 50% of the MVC when smiling^(24)^, and this training was maintained until the end of the program.

The orofacial function training protocols were created in the Biotrainer software, whose configuration allows naming the activity (chewing, swallowing, smiling, resting) and its duration (in the Collection Configuration window, Protocols tab, Activities tab). The activities were then inserted into the Protocol Timeline. The duration of each orofacial function training using electromyographic biofeedback was determined by the speech pathologist (author of this article).

## STATISTICAL ANALYSIS

Data from the four assessments carried out were analyzed using appropriate statistical tests: ANOVA test, Tukey test, Mann Whitney test. In cases where there was a significant interaction between the factors group and time, the analysis of this interaction was predominant over the analysis of isolated factors and the Tukey test was used. The software Statistica 10.0 and SigmaPlot 12.0 were used to analyze the statistical tests, with a significance level of p<0.05 considered in all analyses. The factors group (TG and EBG) and time (assessments T1, T2, T3 and T4) were considered in the analyses of the aspects: facial aging signs and orofacial functions, through inter and intragroup analysis.

## RESULTS

The assessed aspects, regarding aging signs, were registered in a chart - Facial Aging Signs Analysis (FASA), with 56 being the highest score and zero being the lowest. Values were assigned to the frontal, glabellar, periorbital, perioral wrinkles, nasolabial and chin *sulci*, and mandibular and submental contour.

Table 1 shows the descriptive measures of the scores obtained at different moments of the research based on the assessment of the facial aging signs.

**Table 1:** Scores obtained through the assessment of facial aging signs for the therapy group (TG) and electromyographic biofeedback group (EBG) at different assessment moments

	**Minimum**		**Maximum**		**Median**		**Mean**		**SD**	
	**TG**	**EBG**	**TG**	**EBG**	**TG**	**EBG**	**TG**	**EBG**	**TG**	**EBG**
T1	14	14	28	33	22	22	20.80	21.87	4.60	5.01
T2	10	11	36	33	17	19	18.33	20.33	6.58	6.13
T3	15	12	36	32	23	20	23.87	20.93	6.09	5.36
T4	15	11	38	31	23	21	23.20	21.20	6.18	5.39

**Caption: TG=** therapy group; **EBG=** electromyographic biofeedback group; **T1=** 1^st^ assessment; **T2=** 2^nd^ assessment; **T3=** 3^rd^ assessment; **T4=** 4^th^ assessment; **minimum** = lowest score registered in the of Facial Aging Signs Analysis (FASA); **maximum** = highest score registered in AFAS; **median** from the values registered in FASA; **mean and standard** **deviation (SD)** from the values registered in FASA for the facial aging signs variable. **Source:** Developed by the authors.

In the initial T1 assessment, a similar degree of facial aging was observed in the TG and EBG groups, registered in the minimum, maximum and median scores. Numerically different values were found between T1 and T2, with greater improvement in TG and lesser in EBG. The higher total scores in the third and fourth assessments, T3 and T4, indicated that the interruption of the exercises and instructions during washout resulted in increased facial aging signs in both groups.

The two-way repeated measures ANOVA test revealed that, in relation to facial aging signs, there was no statistically significant difference in the group factor (p=0.81), i.e., the TG and EBG scores were similar, regardless of the training carried out with electromyographic biofeedback. However, there was a statistically significant difference in the time factor (p<0.001) and in the interaction between the time and group factors (p<0.001), so the Tukey test was used (Chart 1).

**Chart 1:** Repeated Measures Analysis of Variance (ANOVA) facial aging signs

	SS	Degr. of	MS	F	p
Intercept	54528.03	1	54528.03	468.5641	0.000000
GROUP	6.53	1	6.53	0.0561	0.814427
Error	3258.43	28	116.37		
**TIME**	**176.63**	**3**	**58.88**	**12.8851**	**0.000001**
**TIME/GROUP**	**126.53**	**3**	**42.18**	**9.2304**	**0.000024**
Error	383.83	84	4.57		

**Caption: SS:** sums of squares; **degr of**: degrees of freedom; **MS**: mean square; **F:** F-statistic; **p:** p-value. **Source:** Developed by the authors

As shown in Table 2, the Tukey test indicated that, for the EBG, no statistically significant difference was found between the values attributed to the aging signs (letters a, b, c in T1, T2, T3 and T4), at the four assessment times T1 (21.87), T2 (20.33), T3 (20.93) and T4 (21.20), i.e., the participants did not show a statistically significant change with the intervention proposed. Regarding the TG group, the value registered in the first assessment T1 (20.80, letter a) was statistically higher than that registered in the second assessment T2 (18.33, letter c), indicating a decrease in the aging signs, after carrying out the nine initial sessions. The value obtained in the third assessment T3 (23.87, letter b) was significantly higher than those of the assessments in T1 and T2, i.e., there was an increase of facial aging signs after the washout period. No statistically significant difference was found between the values of the fourth assessment T4 (23.20, letters a, b) and those of the first and third assessments, indicating that there was no improvement, in relation to this aspect, after the six sessions held monthly.

**Table 2:** Analysis of the interaction between time and group factors in relation to facial aging signs - inter-group and intra-group

**Group**	**Time**	**Mean**	**SD**	**Significance** *
EBG	T1	21.87	5.01	a,b,c
EBG	T2	20.33	6.13	a,b,c
EBG	T3	20.93	5.36	a,b,c
EBG	T4	21.20	5.39	a,b,c
TG	T1	20.80	4.60	a
TG	T2	18.33	6.58	c
TG	T3	23.87	6.09	b
TG	T4	23.20	6.18	a,b

Tukey Test (p<0.05)

* **Significance**: different letters indicate a statistically significant difference

**Caption: EBG**= electromyographic biofeedback group; **TG**= therapy group; **T1**= 1^st^ assessment; **T2**= 2^nd^ assessment; **T3**= 3^rd^ assessment; **T4**= 4t^h^ assessment; **Mean** for the variable facial aging signs; **SD**=standard deviation. **Source:** Developed by the authors

**Figure 3:**
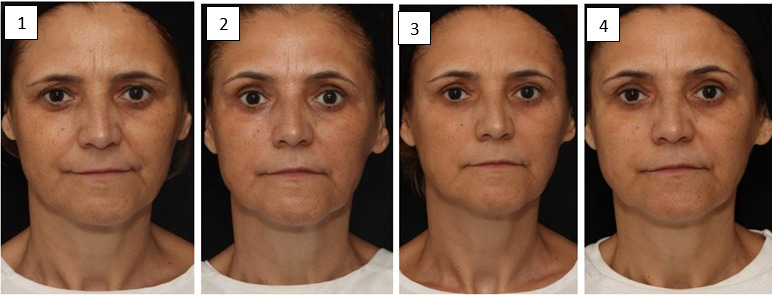
**assessment 1**: Presence of glabellar and periorbital wrinkles, pronounced nasolabial sulcus, moderate mentolabial sulcus; **assessment 2**: These signs were reduced; **assessment 3**: Worsening in glabellar and periorbital wrinkles, slight worsening in nasolabial and mentolabial sulcus; **assessment 4**: Slight improvement in the eye region and left nasolabial sulcus

Figure 3 shows the changes that occurred in each assessment carried out.

Table 3 shows the descriptive measures related to the scores obtained in the orofacial function assessments (chewing, swallowing and speech) at different moments.

**Table 3:** Scores obtained through the assessment of orofacial functions (chewing, swallowing, and speech) for therapy group (TG) and electromyographic biofeedback group (EBG) at different assessment moments

	**Minimum**		**Maximum**		**Median**		**Mean**		**SD**	
	**TG**	**EBG**	**TG**	**EBG**	**TG**	**EBG**	**TG**	**EBG**	**TG**	**EBG**
T1	5	0	16	13	14	8	11.60	8.00	3.89	3.21
T2	2	0	14	12	7	4	6.67	4.27	3.42	3.28
T3	0	0	13	11	3	4	3.93	4.73	3.37	2.81
T4	0	0	8	12	3	5	3.27	4.67	2.05	2.97

**Caption: TG**= therapy group; **EBG**= electromyographic biofeedback group; **T1**= 1^st^ assessment; **T2**= 2^nd^ assessment; **T3**= 3^rd^ assessment; **T4**= 4t^h^ assessment; **minimum**= lowest score registered in the MBGR Protocol; **maximum**= highest score registered in the MBGR Protocol; **median** of the scores registered in the MBGR Protocol; **mean** and **standard deviation** (SD) of the values registered in the MBGR Protocol for the variable orofacial functions. **Source:** Developed by the authors.

The maximum scores of the two groups at T1 indicated that the participants had slight changes in the orofacial functions, considering that the maximum possible score of the MBGR Protocol for analyzing this aspect was 66. At T1, it was observed that the TG had higher scores, i.e., worse performance in the initial assessment of the orofacial functions. The scores obtained in the subsequent assessments, T2, T3, T4, indicated that both groups benefited from the proposed program. It was found that there was a reduction in scores at T2 and stability of the scores until the completion of the program.

Regarding orofacial functions, the two-way repeated measures ANOVA showed no statistically significant difference between the TG and EBG groups (p=0.27), i.e., the scores for these functions were similar, regardless of the training with electromyographic biofeedback. A statistically significant difference was found related to the time factor (p<0.001) and the interaction of time and group factors (p<0.001) and the Tukey test was used (Chart 2).

**Chart 2:** Repeated Measures Analysis of Variance (ANOVA) in Orofacial Functions

	SS	Degr. of	MS	F	p
Intercept	4165.408	1	4165.408	193.4935	0.000000
GROUP	27.075	1	27.075	1.2577	0.27161
Error	602.767	28	21.527		
TIME	647.692	3	215.897	34.9272	0.00000
**TIME/GROUP**	**132.825**	**3**	**44.275**	**7.1627**	**0.00024**
Error	519.233	84	6.181		

**Caption: SS:***sums of squares*; **degr of**: *degrees of freedom*; **MS**: *mean square*; **F:***F-statistic*; **p:** p-value. **Source:** Developed by the authors.

As shown in Table 4, using the Tukey test, both groups showed a significant drop in the value alterations in orofacial functions between the first and second assessment (in EBG, letters c, d in T1, letters a, b in T2 ; in TG letter d in T1 and letters b, c in T2), indicating improvement in orofacial functions after the initial nine sessions, a condition that remained stable in the third and fourth assessments (in EBG, letters a, b in T3, T4; in TG letters a, b in T3, letter a in T4, revealing that there were no significant differences between these times) after the washout period and six monthly sessions, respectively. However, for the TG, a statistically significant difference was found between the second T2 (6.67) and fourth T4 (3.27) assessment (letters b,c in T2, letter a in T4) with a drop in the value in T4, suggesting a positive result after the six monthly sessions.

**Table 4:** Analysis of the Interaction between Time and Group factors in relation to orofacial functions inter-group and intra-group

**Group**	**Time**	**Mean**	**SD**	Significance^*^
EBG	T 1	8.00	3.21	c,d
EBG	T2	4.27	3.28	a,b
EBG	T3	4.73	2.81	a,b
EBG	T4	4. 67	2.97	a,b
TG	T1	11.60	3.89	d
TG	T2	6.67	3.42	b,c
TG	T3	3.93	3.37	a,b
TG	T4	3.27	2.05	a

Tukey Test (p<0.05)

* **Significance**: different letters indicate a statistically significant difference

**Caption: EBG**= electromyographic biofeedback group; **TG**= therapy group; **T1**= 1^st^ assessment; **T2**= 2^nd^ assessment; **T3**= 3^rd^ assessment; **T4**= 4^th^ assessment; **Mean** for the variable facial aging signs; **SD**=standard deviation. **Source:** Developed by the authors.

The Satisfaction Questionnaire, with a maximum score of 90, was responded to by the participants after the initial nine sessions of the proposed program, before viewing their photographic and video documentation recorded in the assessments T1 and T2. As there was no normal distribution of data, the values registered in this questionnaire were analyzed using the Mann-Whitney test (Table 5).

**Table 5:** Analysis of the Level of Satisfaction among participants regarding the proposed therapeutic program – inter-group

	**n**	**Missing**	**Q1**	**Median**	**Q3**
EBG	15	0	82.50	90.00	90.00
TG	15	0	73.50	76.00	80.00

Mann-Whitney Test p<0.001

**Caption: EBG**= electromyographic biofeedback group; **TG**= therapy group; **n**= number of participants; **missing** = number of dropouts; **Q1**= first quartile; **Q3**= third quartile. **Source:** Developed by the authors

A high level of satisfaction was found in both groups. There was a statistically significant difference in the median values of the two groups (p<0.001), with the EBG's level of satisfaction being higher than that observed in the TG; confirming the positive effect of training with electromyographic biofeedback in this aspect. It was found that the use of electromyographic biofeedback was perceived by group participants as an efficient resource, complementary to the orofacial myofunctional training. Chart 3 shows some reports on the positive effect of electromyographic biofeedback.

**Chart 3:** Comments of the BFG participants about electromyographic biofeedback training

**Participants**	**Comments**
1	"Excellent training. I learned how to eat and drink in a way that makes the tongue move correctly."
2	"This technique was essential. Perception with the naked eye without seeing the computer graph is subjective. Once I see the graph (on the screen), it becomes easier to correct improper movements."
3	"The biofeedback served as a parameter to remember and recall the movements that produce oral-aesthetic health."
4	"It was extremely helpful. It provides parameters whether I am working the muscles that were necessary or not."
5	"It greatly helps in the awareness of movement and rhythm."
6	"It helps in confirming that the exercises are being done correctly."
7	"Despite the discomfort from the electrodes, seeing the response to movement in real-time on the screen was spectacular. Understanding the involved muscles and their linked responses was impactful."
8	"It is important to re-educate the brain in new ways to use facial expressions and facial muscle movements. It gave me a new self-awareness about automatic actions that we perform erroneously and unknowingly."
9	"Excellent method because it greatly helped integrate automatic movements in relation to chewing, and the perception of lips and tongue at the time of swallowing."
10	"The training helped me to fix the movements of the tongue and bite. When I saw these movements on the screen, I found it easier to perform them at home."
11	"Visual memory enhances the repetition of exercises at home."
12	"This training helps us to see the act of chewing and swallowing and how it can be improved."
13	"Very interesting. I had difficulty performing some movements and also controlling the facial muscles, but I believe that over time I will overcome all difficulties."
14	"Biofeedback is very helpful in disassociating movements and also in seeing the correct performance."
15	“The training helped me to see how exactly the exercise/movement should be done; it’s imprinted in my memory to use during the routines of my meals and movements.”

**Caption: TG**= therapy group; **EBG**= electromyographic biofeedback group.

**Source**: Developed by the authors.

## DISCUSSION

The number of speech pathologists working in the field of facial aesthetics has increased in recent years. However, no randomized clinical trial publications were found on this topic in Speech Therapy. Studies in this field of activity are scarce and, for the most part, presented descriptions with a small number of subjects and methodological flaws, which makes it impossible to reproduce the proposed procedures^(10)^.

Randomized clinical trials are considered a reference standard as a method for investigating and proving efficacy but are rare in the area of Orofacial Motricity. The present study is the first randomized clinical trial in which electromyographic biofeedback was used in the field of Speech Therapy in facial aesthetics. In this study there was no dropout of participants, and the positive adherence can be justified by the rigorous selection process, effective clarification of the methodological procedures involved and clear rules on the need for daily execution of the guidelines prescribed in therapy, as well as permanence at the different stages of the research.

Statistical tests showed that the majority of participants in this research presented minor alterations of assessed facial aging signs, differently from what was expected in the age group studied – 50 to 60 years. It is presumed that the healthy lifestyle predominant in the group of volunteers, as well as their preferences for adopting natural aesthetic care, explain these results.

The total score measured showed that only the TG group had a significant change of facial aging signs. In this group, the scores registered in the second, third and fourth assessments showed, respectively, improvement in facial aging signs after carrying out the initial nine sessions, worsening after an interruption of eight weeks, practically no change after the six monthly sessions, with improvement only in mandibular ptosis and static frontal wrinkles. For the EBG group, in the final period of six months, there was an improvement in smile function, with consequent reduction of dynamic periorbital wrinkles. However, these aesthetic improvements were considered minimal in view of the many aspects covered in the treatment.

Once no worsening was found in the chewing and swallowing patterns learned by participants in both groups, the score result with greater loss in terms of aesthetic aspects in the six-month period after the washout could be related both to the interruption of the use of the Transpore^TM^ inelastic bandage, facial and tongue exercisers, tourniquet, cork exercise, as well as the likely discouragement of the participants in the second stage of the intervention, with the observation of aesthetic losses presented after the third evaluation.

The relationship between facial aging signs and excessive contraction of facial muscles has been described by several professionals. For some speech pathologists, there would be a reduction in the facial aging signs with adjustment of orofacial functions^(5-7)^. The analysis of the participants’ performance regarding the changes that occurred in the assessed orofacial functions, confirmed by the statistical tests applied, made it possible to question this relationship. In this study, improvement and stability of the chewing and swallowing patterns learned were observed until the study was completed; it can be stated that these patterns were automated, without a corresponding change in the facial aging signs, whose scores increased after the washout. That is, in none of the assessed aesthetic aspects was there a relation observed with the improvement in orofacial functions.

In the publications consulted this correlation was reported in a generic way, i.e., without reference to the detection of an altered aspect in the assessment that has been corrected with a specific intervention^(5-7)^. Thus, it can be stated that the scores for facial aging signs, indicating improvement in the second assessment T2 (mainly static frontal wrinkles) and worsening of signs in assessment T3 (periorbital and static perioral wrinkles, submental ptosis and mentolabial sulcus), refer to the effect of specific procedures carried out in the first stage of the proposed program and their interruption during the washout period, when all the materials used in therapy and the sheet containing the prescribed guidelines for carrying out the exercises and functional training at home were collected. In this respect, the procedures used proved to be efficient in reducing wrinkles and aging signs in the upper third of the face. Speech therapy intervention proved to be an alternative to mitigate facial aging signs, in addition to existing dermatological procedures to reduce excessive contraction of mimic muscles^(9)^.

In the 10^th^ week of this study, the participants responded to the Satisfaction Questionnaire, which contained questions related to the therapeutic program, such as: level of satisfaction with the results obtained, expectations, goals achieved, therapist performance and possibility of recommending this program to other individuals. The statistical results showed that the participants in both groups were satisfied with the results of the program, with a higher level of satisfaction in the EBG group.

Evaluating the participants’ satisfaction with the program was an important procedure, considering that dissatisfaction with appearance is the factor that guides the client in the search for a facial aesthetic intervention^(26)^. Aesthetic treatments have a positive impact on the emotional health of individuals. Many seek minimally invasive treatments to combat facial aging and promote a natural, relaxed appearance^(26)^.

Unlike the present study, in which the participants answered the questionnaire before comparing the photographic and video documentation carried out in the first and second assessments, in most publications in which individuals’ satisfaction was investigated, the self-evaluation was carried out through comparison between photographic images taken before and after aesthetic procedures^(26-28)^.

The results measured in the MBGR protocol and the FASA showed the persistence of functional alterations and facial aging signs even after the initial nine weeks of intervention. However, the Satisfaction Questionnaire showed that there was a positive response from participants regarding the orofacial and aesthetic myofunctional treatment received. This divergence between the results observed after the intervention and the level of satisfaction of the participants was also highlighted in a survey conducted by plastic surgeons^( 27))^.

In the present study, a high level of satisfaction was found regarding the non-invasive procedures used, which promoted slight aesthetic changes and greater functional changes. This result corroborates observations by authors who consider that subtle changes in appearance can substantially affect individuals’ self-esteem and happiness^(28)^.

The present study is the first randomized clinical trial in the field of Speech Therapy in facial aesthetics using electromyographic biofeedback. Some researchers stated that one of the advantages of electromyographic biofeedback would be the visualization of muscular activity on the computer screen, allowing patients to participate more actively with greater control over the orofacial muscles^(15,16)^. In this study, the answers obtained in the Satisfaction Questionnaire confirmed this advantage: the EBG participants were able to learn to control the recruitment of the muscles worked during chewing, swallowing, and smiling training. It is worth remembering that the electrodes positioned in the risorius region, for smile training, do not prevent the capture of the electrical signal from the adjacent zygomaticus major, minor and buccinator muscles (cross talk). Even so, the participants were able to compare the contraction of the muscles in this region and the lower orbital portion of the *orbicularis oculi* muscle and, through voluntary control and visualization of the signal on the computer screen, reduce muscle contraction in the eye region when smiling, with consequent reduction of static and dynamic periorbital wrinkles.

The positive effects of this resource to reduce the contraction of the muscles involved in chewing, swallowing and articulating speech and, consequently, reducing facial wrinkles have been described, to date, in a single publication on the use of electromyographic biofeedback in Speech Therapy in facial aesthetics^(14)^.

A hypothesis formulated in this study was that EBG participants would obtain higher scores than those in the TG. However, there was no significant difference when comparing performance between the two groups. Both showed improvement and maintenance of chewing and swallowing patterns learned, without changes in smile and speech.

Regarding facial aging signs, the TG and EBG scores were similar at the different moments of assessment, regardless of the training carried out with electromyographic biofeedback.

There was a difference between the groups in the level of satisfaction regarding the results obtained after completing the first stage of the program, with EBG having a higher level of satisfaction. The participants reported that viewing the image corresponding to the trained muscular activity on the computer screen favored awareness, control and learning of the movements necessary to carry out appropriate patterns of chewing, swallowing and facial expression during the sessions and the consolidation of these patterns in their homes. The positive impact provided by visual feedback was equally reported by healthy and dysphagic subjects in another study in the area of dysphagia^(16)^.

Despite the agreement that training with electromyographic biofeedback improved muscle contraction in individuals with some orofacial myofunctional alterations, to date, no studies have been found proving differences between the final outcomes of interventions carried out with and without the association of this therapeutic resource^(15)^.

The washout, an uncommon resource in speech therapy research^(^^29^^)^, was important in this study to confirm losses after the interruption of the therapeutic program. One of the hypotheses formulated in the present study was that there would be functional and aesthetic losses with the interruption of the exercises. This hypothesis was not confirmed in relation to the chewing and swallowing functions, since the patterns learned in the first stage of the proposed program were integrated, nor in relation to the speech and smile functions, whose scores remained practically unchanged from the beginning to the end of the research. However, there was aesthetic loss and worsening of static perioral and periorbital wrinkles, and mandibular ptosis for the TG participants, and worsening of the mentolabial sulcus and static frontal wrinkles in both groups, confirming that the participants did not perform the exercises during this period. The interruption of the proposed exercises was probably the cause of these aesthetic losses. Since aging is inexorable, it is suggested that the routine performance of specific Speech Therapy exercises may be beneficial to reduce some facial aging signs.

The results of the present study suggest that, unlike what has been pointed out by studies published to date^(6,7)^, although inadequate, the intermittent contraction of the perioral muscles during chewing, swallowing and speaking would not necessarily result in a higher incidence of wrinkles in this region. We can partially agree with these studies regarding the correlation between reduced exaggerated contraction of mimic muscles and reduced facial wrinkles. In this study, this association was found specifically in relation to static frontal wrinkles. However, the adequacy of orofacial functions is considered an important factor in promoting healthy aging, regardless of their impact on facial aesthetics.

The proposal to carry out a second stage of the study was based on the hypothesis that the interruption of exercises during the washout period would lead to aesthetic and functional losses, which would be overcome with the six sessions carried out monthly. No research was found in the area of Speech Therapy that presented a similar proposal.

Regarding orofacial functions, the alterations observed in the initial assessment were practically overcome in the first stage of the program, with no worsening of chewing and swallowing patterns learned after the washout period in both groups.

Regarding facial aging signs, a drop in scores was observed in some aspects, which indicated positive effects of this proposal, after the six monthly sessions. For the TG there was an improvement and reduction in mandibular ptosis and static frontal wrinkles, while for the EBG there was an improvement in the smile function, i.e., a reduction in the dynamic periorbital wrinkles. Considering the increase in scores in almost all aspects assessed after the washout period, especially in the TG, the aesthetic changes observed in the second stage of the program were not significant. Considering that facial aging is progressive, it is suggested that speech therapy procedures to reduce aging signs be maintained without interruption.

This study brought relevant contributions to Speech Therapy, both in the area of Orofacial Motricity and of Facial Aesthetics. It was found that the functional training to adapt the orofacial functions of chewing and swallowing, carried out in nine sessions, was sufficient for the motor patterns learned to be integrated/automated, with no functional losses after the washout period; the program tested proved to be effective in reducing wrinkles and facial aging signs; the results presented showed the possibilities and limitations of this program and, in this form, point the way to future research in the field of Speech Therapy in facial aesthetics, where scientific publications are scarce.

### Limitations of the study

As previously mentioned, most participants of this study showed slight alterations in facial aging signs assessed. Therefore, new studies may apply the program to women over 60 years of age. It is also suggested, in future studies, to include a control group and avoid presenting partial results to the participants (comparing the first assessment with the second) before carrying out the third and fourth assessments, thus preventing interference in the participants’ assessment of the treatment carried out based on the information obtained.

The proposed intervention in the present study encompassed only one of the structures that change with aging, that is, the facial muscles. It is believed that more positive results could be achieved by combining speech therapy with other treatments, such as treatments proposed by dermatologists, whose procedures, more or less invasive, include interventions to repair the structural changes inherent to facial aging.

## CONCLUSION

The proposed program resulted in aesthetic changes, mitigation of facial aging signs, especially for participants in the TG, and functional changes in facial expression, adequacy of chewing and swallowing, no changes in speech and smile functions, for all research participants (TG and EBG groups), after the nine weekly sessions. Interrupting the program for eight weeks resulted in aesthetic losses, but not functional losses, for the TG and EBG groups. The use of electromyographic biofeedback resulted in higher scores for the EBG group, compared to the TG, only in relation to the level of satisfaction, having no impact on the training of chewing, swallowing, and smiling patterns. The participants’ level of satisfaction was higher than the results measured in the MBGR Protocol and FASA. The six monthly sessions after the washout period had limited effect in overcoming aesthetic losses.
