# Supplementary material for: Effectiveness of orofacial myofunctional intervention to mitigate facial aging signs: a clinical trial
Source: Codas. 2024 Aug 19;36(5):e20230016. doi: 10.1590/2317-1782/20242023016en (PMC11340872; doi:10.1590/2317-1782/20242023016en)
Supplement: Translation

# Translation: Efetividade da intervenção miofuncional orofacial para atenuar sinais do envelhecimento facial: ensaio clínico

**DOI:** 10.1590/2317-1782/20242023016pt

**Authors:** Yasmin Salles Frazão, Silvia Bertacci Manzi, Lilian Krakauer, Giédre Berretin-Felix

**Affiliations:** 1 Programa de Pós-graduação em Fonoaudiologia, Faculdade de Odontologia de Bauru, Universidade de São Paulo – USP - Bauru (SP), Brasil.; 2 Conselho Federal de Fonoaudiologia – CFFa - São Paulo (SP) Brasil.; 3 Pontifícia Universidade Católica de São Paulo – PUCSP - São Paulo (SP), Brasil.; 4 Departamento de Fonoaudiologia, Faculdade de Odontologia de Bauru, Universidade de São Paulo – USP - Bauru (SP), Brasil.

**Keywords:** Estética, Fonoaudiologia, Terapia Miofuncional, Eletromiografia, Rejuvenescimento, Envelhecimento

## Abstract

**Objetivo:**

Propor e verificar a eficiência de um programa de intervenção miofuncional para atenuar sinais do envelhecimento facial e equilibrar as funções orofaciais.

**Método:**

30 mulheres, entre 50 e 60 anos, divididas aleatoriamente em: grupo terapia (GT), submetido ao programa de terapia miofuncional orofacial e grupo biofeedback eletromiográfico (GBE), submetido ao mesmo programa associado ao biofeedback eletromiográfico para treinamento da mastigação, deglutição e sorriso. Aspectos estéticos e oromiofuncionais foram avaliados a partir da documentação das fotografias e vídeos, do Protocolo de avaliação miofuncional orofacial MBGR e escalas de avaliação dos sinais de envelhecimento facial descritas na literatura. Sessões de 50 minutos foram realizadas semanalmente, durante nove semanas e mensalmente, durante seis meses, após washout. Três avaliações, idênticas à inicial, foram realizadas na décima semana, oitava semana após washout e conclusão da pesquisa. As participantes responderam ao Questionário de Satisfação na décima semana.

**Resultados:**

A análise estatística realizada, por meio dos testes ANOVA, Tukey e Mann Whitney, para comparação inter e intragrupos, demonstrou que: houve atenuação dos sinais do envelhecimento facial, principalmente no GT e equilíbrio das funções mastigação e deglutição nos dois grupos; houve impacto do biofeedback eletromiográfico sobre o grau de satisfação das participantes, sendo maior no GBE; a interrupção do programa durante oito semanas resultou em perdas estéticas, principalmente no GT, mas não em perdas funcionais, nos dois grupos; as seis sessões realizadas mensalmente tiveram impacto limitado para superação das perdas estéticas ocorridas após washout.

**Conclusão:**

O programa proposto resultou em atenuação dos sinais de envelhecimento, principalmente no grupo GT e melhoria nas funções orofaciais, nos dois grupos.

## INTRODUÇÃO

O envelhecimento facial é resultado de diversos fatores inerentes ao processo natural do envelhecimento e, também, de fatores relacionados ao estilo de vida dos indivíduos, que pode potencializar o aparecimento de rugas e sulcos faciais, tais como exposição ao sol sem uso de protetor solar^(1,2)^, hábitos deletérios, como tabagismo, alimentação não balanceada e sedentarismo^(1,3)^. A contração exagerada de músculos envolvidos nas funções orofaciais é outro fator frequentemente relatado por médicos e fonoaudiólogos^(4-7)^ e pode resultar em rugas estáticas e/ou dinâmicas, de acordo com a intensidade, frequência e duração destas contrações, assim como das características dentoesqueléticas individuais^(6,8)^.

Diversos profissionais se dedicam à pesquisa e atuação em estética facial utilizando recursos clínicos, mais ou menos invasivos, para atenuar envelhecimento facial. Variadas técnicas cirúrgicas e procedimentos, tais como preenchimento facial, utilização de toxina botulínica e aplicação de laser, são alguns dos recursos propostos por dermatologistas e cirurgiões plásticos^(2,9)^. A terapia miofuncional orofacial proposta por fonoaudiólogos é uma abordagem não invasiva, que atende à demanda de clientes que optam por um envelhecimento natural^(5-7)^.

Constatou-se existir uma grande variedade de recursos clínicos empregados por fonoaudiólogos na intervenção em estética facial. Para alguns, os exercícios de contração muscular, realizados após manobras de relaxamento muscular, seriam essenciais para atenuar as rugas faciais; em oposição a esses, há os que condenaram a utilização de exercícios de contração muscular; outros propuseram a combinação destes exercícios com adequação das funções orofaciais^(10)^ e há, ainda, alguns que utilizaram termoterapia, crioterapia^(11)^ e estimulação elétrica neuromuscular^(12)^. Apesar da eletromiografia de superfície (EMGs) ser considerada um instrumento valioso tanto para o diagnóstico, como para reabilitação de indivíduos com alterações orofaciais miofuncionais decorrentes de diversas etiologias^(13)^, em apenas um estudo o *biofeedback* eletromiográfico foi recurso utilizado na intervenção fonoaudiológica em estética da face^(14)^, havendo consenso, também, entre pesquisadores de outros estudos em que esse recurso foi utilizado^(15,16)^, que tal técnica representa uma modalidade coadjuvante promissora no processo terapêutico.

Na maioria das publicações consultadas^(5-7,11,12)^, os autores descreveram mudanças estéticas significativas e afirmaram que os diversos exercícios empregados atenuaram os sinais de envelhecimento. Entretanto, pode-se questionar as evidências descritas nestes estudos ao se analisar sua metodologia pouco precisa, conforme aponta Valente em revisão sistemática^(10)^. Em sua maioria, foram estudos que apresentaram número reduzido de sujeitos, avaliados pelos próprios pesquisadores, fato que reduziria o grau de confiabilidade destes resultados^(10)^. Tais achados demonstram a necessidade de realização de pesquisas que apresentem maior rigor metodológico e aferição quantitativa dos resultados. O presente estudo é o primeiro ensaio clínico em que um programa terapêutico direcionado à atenuação dos sinais de envelhecimento facial foi testado na íntegra.

Sendo assim, o objetivo da presente pesquisa foi propor e verificar a eficiência de um programa de intervenção miofuncional orofacial para atenuar os sinais de envelhecimento facial e equilibrar as funções orofaciais. Para tanto foi verificado: se haveria perdas estéticas e miofuncionais orofaciais com a interrupção do programa terapêutico (período de *washout*); se os resultados obtidos pelo grupo *biofeedback* eletromiográfico (GBE) seriam superiores aos do grupo terapia (GT); se o grau de satisfação das voluntárias, em relação ao programa terapêutico, seria compatível aos resultados aferidos no protocolo MBGR e na Análise dos sinais de envelhecimento facial (ASEF); se a realização de seis sessões, uma vez ao mês, durante seis meses, seria suficiente para reverter as perdas estéticas e funcionais, após período de *washout.*

## MÉTODO

Trata-se de ensaio clínico randomizado controlado, aprovado pelo Comitê de Ética, parecer sob número 2.235.918 – CAAE: 71680017.0.0000.5417. Todos os participantes foram devidamente informados sobre os objetivos e procedimentos realizados na pesquisa e assinaram o Termo de Consentimento Livre e Esclarecido.

As participantes da pesquisa, do sexo feminino, tinham entre 50 e 60 anos (53,73 ± 2,79). Os critérios de exclusão adotados foram: realização de procedimentos faciais invasivos (cirurgia plástica, preenchimento facial, aplicação de toxina botulínica, aplicação de laser) e não invasivos (drenagem; massagens; medicamentos; novos cremes, distintos dos que habitualmente usava) no ano anterior ao início dos atendimentos e durante participação na pesquisa, histórico de deformidade dentofacial esquelética, disfunção temporomandibular, presença de ronco, ausência de mais de um elemento dentário, intolerância aos alimentos utilizados na avaliação e terapia, indisponibilidade para cumprir o cronograma da pesquisa por 42 semanas.

Por meio de cálculo amostral, realizado a partir de estudo piloto, determinou-se o número de 30 voluntárias, as quais foram distribuídas aleatoriamente, por meio de planilha do programa *Microsoft Office Excel*, em dois grupos: grupo terapia (GT), submetidas ao programa terapia miofuncional orofacial estipulado pelo projeto; grupo *biofeedback* eletromiográfico (GBE), submetidas ao mesmo programa terapêutico complementado por *biofeedback* eletromiográfico, para treinamento da mastigação, deglutição e sorriso.

As participantes foram submetidas a uma avaliação inicial, que incluiu a documentação fotográfica e em vídeo de algumas das provas contidas no Protocolo de Avaliação Miofuncional Orofacial MBGR^(17)^. Para realização desta documentação, as participantes apresentaram-se vestidas com blusa clara, sem gola, sem maquiagem e sem brincos. O padrão de documentação fotográfica e em vídeo foi o proposto por Frazão e Manzi^(18)^. Três avaliações, idênticas à inicial, foram realizadas na 10ª sessão, na oitava semana após *washout* e na conclusão da pesquisa ao final dos seis meses seguintes ao *washout*. As participantes responderam ao Questionário de Satisfação, antes da realização das documentações fotográfica e em vídeo, na décima semana, em papel impresso, sem se identificarem, na ausência da pesquisadora na sala. Os questionários respondidos foram lacrados, colocados em envelope plástico, igualmente lacrado, na presença da participante e, posteriormente, foram entregues às fonoaudiólogas avaliadoras.

Após a avaliação inicial, as participantes dos dois grupos foram submetidas a uma intervenção terapêutica com duração de nove semanas (uma sessão semanal), quando aprenderam os exercícios isotônicos e isométricos (músculos bucinadores, porção palpebral do músculo orbicular do olho e músculos supra-hioideos), os padrões adequados de mastigação, deglutição e de controle da mímica facial durante a comunicação/fala. Após esta etapa inicial, seguiu-se um período de oito semanas em que o programa foi interrompido (*washout*). Nos seis meses seguintes ao *washout*, as sessões foram retomadas e realizadas na frequência de um atendimento ao mês.

As documentações fotográficas e em vídeos, das trinta participantes, nas quatro avaliações realizadas (T1, T2, T3 e T4, totalizando cento e vinte avaliações), foram distribuídas aleatoriamente e enviadas às duas fonoaudiólogas, especialistas em Motricidade Orofacial, com trinta e quatro e trinta e oito anos de experiência clínica, previamente calibradas. A calibração foi concluída quando as avaliadoras atingiram uma porcentagem de confiabilidade mínima de 90%. Os escores do Protocolo MBGR, da Análise dos sinais de envelhecimento facial (ASEF), baseada nas escalas fotonuméricas validadas na literatura^(19-22)^ foram aferidos pelas avaliadoras, que também tabularam os escores registrados no Questionário de Satisfação. As avaliadoras receberam fotos nas posições frontal, 45º e perfil - direito e esquerdo, para a verificação das rugas estáticas, filmagens das funções orofaciais (fala, mastigação e deglutição) e imagens recortadas de vídeo (captura de tela), para avaliação das rugas dinâmicas, que registraram a contração dos músculos: porção frontal do músculo occipitofrontal, corrugador do supercílio, orbicular dos olhos e orbicular da boca. As avaliadoras efetuaram a avaliação cega desta documentação e decidiram, por consenso, nas respostas divergentes, alcançando uma concordância de 92% para o Protocolo MBGR e de 98% para a ASEF. A Figura 1 contém o fluxograma das etapas da pesquisa.

**Figura 1:** Fluxograma das etapas da pesquisa

Na presente pesquisa, iniciada em 2017, foram selecionadas algumas provas contidas no Exame Clínico Miofuncional Orofacial, versão 2014 do Protocolo MBGR: funções orofaciais (mastigação habitual, deglutição habitual de alimento sólido), deglutição dirigida (água), fala; aspectos gerais (deglutição de saliva durante provas de Fala). Neste Protocolo atribui-se escore maior às alterações miofuncionais orofaciais acentuadas e menor às alterações reduzidas. Para a análise dos dados, foi considerada a soma total dos escores das funções de mastigação, deglutição e fala avaliados por meio do Protocolo MBGR.

Uma escala fotonumérica foi elaborada, a partir da seleção de algumas imagens das escalas validadas propostas por Flynn et al.^(19)^, Narins et al.^(20)^, Carruthers et al.^(21)^, Jones et al.^(22)^, para avaliar as rugas e sinais de envelhecimento nos terços faciais superior, médio e inferior, nas quatro avaliações realizadas. De acordo ao preconizado pelos autores destas escalas foram atribuídos valores às rugas estáticas e dinâmicas frontais, glabelares, periorbitárias^(19)^; rugas periorais, ao sulco nasolabial e lábio mentoniano, à ptose em contorno mandibular (frontal) e submandibular (perfil/papada)^(20)^; rugas nas bochechas^(21)^; rugas cervicais^(22)^. O valor zero foi atribuído à ausência de rugas, sulcos e ptose e o valor 4 à presença acentuada desses sinais de envelhecimento. Os valores máximos (56) e mínimo (zero) atribuídos, respectivamente, à maior ou menor presença de sinais de envelhecimento facial, foram registrados em um quadro - Análise dos sinais de envelhecimento facial (ASEF), sendo a soma total dos escores dos aspectos estéticos avaliados, o valor numérico considerado para a análise estatística dos dados.

O Questionário de Satisfação, com pontuação máxima de 90, foi respondido pelas participantes, após as nove sessões iniciais do programa proposto, antes de visualizarem suas documentações fotográficas e em vídeo, registradas nas avaliações T1 e T2. A soma total dos valores referentes às respostas das participantes foi considerada para a análise estatística dos dados.

## PROGRAMA TERAPÊUTICO: TERAPIA MIOFUNCIONAL OROFACIAL PARA ATENUAR OS SINAIS DE ENVELHECIMENTO FACIAL (TMOEF)

O programa terapêutico foi constituído de duas etapas: na primeira foram realizadas nove sessões, semanalmente; na segunda foram realizadas seis sessões, mensalmente, iniciadas após período de *washout*. As participantes dos dois grupos se submeteram ao mesmo programa terapêutico. A coleta de sinais e o treinamento funcional da mastigação, deglutição e sorriso, para os participantes do GBE, serão descritos no item – **Treino com *Biofeedback* Eletromiográfico** **– grupo GBE.**

Os objetivos terapêuticos foram trabalhados durante as sessões realizadas nas duas etapas do programa terapêutico, por meio das estratégias pertinentes. Os exercícios foram apresentados paulatinamente a cada sessão e, ao final das sessões, as participantes recebiam, em papel impresso, as orientações atualizadas que deveriam ser realizadas diariamente em suas residências e entregavam para a pesquisadora a folha impressa contendo as orientações anteriores. Apesar de não ter havido um controle de registro da execução das orientações em domicílio, a pesquisadora pôde constatar as mudanças nos padrões motores treinados a cada semana. A realização efetiva dos exercícios prescritos para casa foi constatada pela melhoria no desempenho na execução de cada exercício a cada sessão. Todos os procedimentos utilizados no programa terapêutico foram descritos detalhadamente para garantir que este estudo possa ser reproduzido e utilizado por outros pesquisadores, podendo ser acessado no site da Biblioteca Digital de Teses e Dissertações da USP^(23)^.

Os seguintes objetivos e estratégias, que serão descritos resumidamente, foram executados em terapia: alongamento manual dos músculos temporal e masseter direito e esquerdo, com objetivo de diminuir sua contração excessiva; manutenção, durante o dia, de dois centímetros de garrote, com cinco milímetros de diâmetro no vestíbulo oral (intersecção entre mentual e lábio inferior), com o objetivo de reduzir a contração da região perioral. Uso da bandagem inelástica Transpore^TM^ (Figura 2).

**Figura 2:** Colocação da bandagem inelástica - Transpore™ - na testa e glabela (Transpore^TM^ com largura de 25 mm), glabela em V e canto externo dos olhos (Transpore^TM^ com largura de 12 mm). Colocação de fita dupla (uma sobre outra) em todas as áreas (testa, glabela e canto externo dos olhos)

durante o período de sono noturno, sobre os músculos occipito frontal, corrugador do supercílio e orbicular do olho (canto externo dos olhos), para diminuir a contração dos músculos do terço superior e médio da face; as participantes foram treinadas em terapia a colocar o Transpore^TM^ adequadamente e, também, registraram, em seus celulares, a fotografia da bandagem inelástica devidamente posicionada em suas faces. Realização de exercícios isométricos e isotônicos para condicionar os músculos da região das bochechas, os supra-hioideos, os linguais e atenuar sulco nasogeniano, ptose de bochechas e ptose submandibular, utilizando os exercitadores Facial Plus e Lingual – Pró-Fono^®^. Realização de exercícios isotônicos e de resistência para condicionar a porção palpebral do músculo orbicular do olho, com o objetivo de manter olhos mais abertos, atenuar a contração dos músculos frontal, corrugador do supercílio e orbicular do olho durante comunicação. Exercício de falar com rolha de vinho entre os dentes (“fala em rolha”), mantendo a abertura da boca pelo diâmetro da rolha, como técnica para atenuar a contração excessiva dos músculos periorais na articulação da fala. Realização de treinamento funcional para adequar os padrões de mastigação, deglutição, com alimento líquido, sólido e bala Halls^®^, o sorriso suave (“sorriso social”), sem selamento labial e controle da mímica facial durante a comunicação.

Na décima semana, quando realizada a segunda avaliação, as participantes devolveram a folha impressa com as orientações, assim como todos os materiais utilizados durante as nove sessões (exercitadores facial e lingual, garrote, rolha de vinho, bandagem inelástica Transpore^TM^ e bala Halls^®^); o recolhimento de todos os materiais e da folha impressa contendo as orientações prescritas para realização dos exercícios em domicílio foi o procedimento adotado para garantir que os exercícios não fossem executados durante o período de *washout.* Os materiais foram guardados em sacos plásticos individuais (exceto a bala Halls^®^, que foi descartada; uma nova caixa de balas foi dada aos participantes após o *washout*), identificados com nome da participante, sendo posteriormente devolvidos, na segunda etapa do programa.

A segunda etapa do programa terapêutico iniciou-se após o período de oito semanas de *washout*, quando as participantes foram avaliadas (terceira avaliação) e iniciaram a sequência de seis atendimentos realizados mensalmente. Nesta ocasião, foi efetuada a revisão dos exercícios e orientações, que deveriam ser praticados pelas participantes diariamente em suas residências; as orientações entregues em folha impressa na primeira sessão após o *washout* foram as mesmas recebidas na nona sessão. Ao final dos seis meses, as participantes receberam as seguintes orientações finais, em folha impressa, com a recomendação de realizá-las diariamente, por toda vida: utilização da bandagem inelástica Transpore^TM^, execução dos exercícios isotônicos e isométricos para olhos, região de bochechas (exercitador facial), língua e músculos supra-hioideos (exercitador lingual); manutenção do controle funcional da mastigação, deglutição e fala, porém, sem a repetição do treinamento funcional inicial. Receberam a orientação para executarem a manobra de pressão na intersecção entre o músculo mentual e lábio inferior, em substituição ao uso do garrote e alongamento dos músculos masseter e temporal quando necessário, isto é, quando perceberem contração muscular exagerada nestas regiões faciais.

## TREINO COM *BIOFEEDBACK* ELETROMIOGRÁFICO – GRUPO GBE

A coleta de sinais e treinamento funcional da mastigação, deglutição e sorriso, para os participantes do GBE, foi realizado com o *software* Biotrainer no aparelho New Miotool Face, da Miotec, que possui oito canais conectados a sensores ativos diferenciais, com resolução de 16bits, frequência de amostragem 2kHz, filtro passa-baixa 20Hz, filtro passa-alta 500Hz, *notch* 60Hz, com conexão de garras e um de referência (terra). Para a captação do sinal elétrico, realizado com o mesmo aparelho, foram utilizados eletrodos duplos passivos diferenciais, Double Trace LH-ED4020; dimensões: 44 mm de comprimento, 21 mm de largura, 20 mm de centro a centro, colocados sobre músculos envolvidos na mastigação, deglutição e sorriso (masseter direito e esquerdo; região de zigomático maior, menor, risório; orbicular da boca – lábio superior; supra-hioideos; porção orbital inferior do músculo orbicular do olho). O eletrodo de referência (terra) foi posicionado sobre o processo estiloide da ulna do braço direito dos sujeitos. As áreas onde foram posicionados os eletrodos, foram previamente higienizadas com gaze e álcool 70º, em todas as sessões em que o treino com *biofeedback* eletromiográfico foi realizado.

A contração voluntária máxima (CVM) dos músculos selecionados foi aferida, e o valor de 50% da CVM foi o parâmetro estabelecido para o aumento ou diminuição da contração muscular durante os treinos, isto é, atenuar contração dos músculos masseteres, orbicular da boca, zigomáticos maior e menor, orbicular dos olhos; aumentar contração dos músculos supra-hioideos. O percentual da intensidade da contração muscular pode ser estabelecido no software, na janela Configuração de Coleta, aba Protocolos, aba Atividades, janela Nova Atividade, Intensidade (%). As participantes foram orientadas a aumentar a contração muscular, ultrapassando o traçado-alvo ou reduzir a contração muscular mantendo-se abaixo deste traçado.

Para o treinamento com *biofeedback* eletromiográfico, os eletrodos foram colocados em uma sequência para favorecer o controle progressivo da cliente sobre os movimentos dos diferentes grupos musculares na execução das funções orofaciais treinadas.

Assim, para treino da mastigação unilateral alternada, iniciado na segunda sessão, eletrodos foram posicionados, paralelamente ao sentido da fibra muscular, sobre músculos masseter direito e esquerdo e as participantes foram orientadas a mastigar uma porção na íntegra de um lado, outra porção na íntegra do outro lado; na terceira e quarta sessões, os eletrodos foram posicionados sobre músculos masseter direito e esquerdo e músculo orbicular da boca (lábio superior), com exigência de contração labial abaixo da média de 50% da CVM^(24)^, para atenuar a possibilidade de formação de rugas dinâmicas e estáticas na região perioral; finalmente, para o treino de mastigação bilateral alternada, em que uma mesma porção é mastigada ora de um lado, ora do outro lado, realizado da quinta à nona sessão, os eletrodos foram posicionados sobre os músculos masseter direito e esquerdo, músculo orbicular da boca (lábio superior) e região de músculos supra-hioideos, uma vez que, nestas sessões, o treinamento da mastigação foi associado ao da deglutição de uva passa triturada e as participantes foram orientadas a contrair os músculos supra-hioideos acima da média de 50% da CVM ao deglutirem.

Para o treino da deglutição, iniciado na terceira sessão com deglutição de alimento pastoso (iogurte grego), eletrodos foram posicionados, na região dos músculos supra-hioideos; na quarta e quinta sessões (deglutição de água), os eletrodos foram posicionados na região dos músculos supra-hioideos, músculo orbicular da boca (lábio superior); finalmente, para realização do treinamento de deglutição de uva passa triturada, da quinta à nona sessão, os eletrodos foram posicionados na região dos músculos supra-hioideos, orbicular da boca (lábio superior) e masseter direito e esquerdo. Durante a deglutição, as participantes foram orientadas a contrair os músculos supra-hioideos acima da média de 50% da CVM, com consequente aumento da contração dos músculos da língua e maior força de ejeção^(25)^ e manter uma contração reduzida dos músculos masseteres e lábio superior, apenas o suficiente para estabilizar a mandíbula e manter lábios fechados sem tensão.

No treino do sorriso, iniciado na quinta sessão, eletrodos foram posicionados, inicialmente, na região dos músculos risório esquerdo e direito, com orientação às participantes que fizessem um sorriso suave, sem selamento labial, enquanto pensavam em algo alegre; posteriormente, da sexta à nona sessão, eletrodos foram posicionados na região dos músculos risório esquerdo e direito e na porção orbital inferior do músculo orbicular dos olhos direito e esquerdo, com orientação de manterem a contração do orbicular dos olhos abaixo da média de 50% da CVM ao sorrir^(24)^, treino mantido até o final do programa.

Os protocolos de treinamento das funções orofaciais foram criados no *software* Biotrainer, cuja configuração permite nomear a atividade (mastigação, deglutição, sorriso, repouso) e sua duração (na janela Configuração de Coleta, aba Protocolos, aba Atividades). As atividades são, então, inseridas na Linha do Tempo do Protocolo. A duração do treinamento de cada uma das funções orofaciais utilizando o *biofeedback* eletromiográfico foi determinada pela terapeuta (autora do presente artigo).

## ANÁLISE ESTATÍSTICA

Os dados das quatro avaliações realizadas foram analisados por meio de testes estatísticos pertinentes: teste ANOVA, teste *Tukey,* teste *Mann Whitney.* Nos casos, em que houve interação significativa entre os fatores grupo e tempo, a análise desta interação foi predominante sobre a análise dos fatores isolados e o teste Tukey foi utilizado. Os *softwares* *Statistica* 10.0 e *Sigma Plot* 12.0 foram utilizados para análise dos testes estatísticos, sendo o nível de significância p<0,05 considerado em todas as análises. Os fatores grupo (GT e GBE) e tempo (avaliações T1, T2, T3 e T4) foram os considerados nas análises dos aspectos: sinais de envelhecimento facial e funções orofaciais, por meio de análise inter e intragrupos.

## RESULTADOS

Os aspectos avaliados, referentes aos sinais de envelhecimento, foram registrados em um quadro - Análise dos Sinais do Envelhecimento Facial (ASEF), sendo 56 o maior escore e zero o menor. Foram atribuídos valores às rugas frontais, glabelares, periorbitárias, periorais, aos sulcos nasolabial e lábio mentoniano, ao contorno mandibular e submandibular (papada).

Na Tabela 1 são apresentadas as medidas descritivas relativas aos escores obtidos, nos diferentes momentos da pesquisa, a partir da avaliação dos sinais de envelhecimento facial.

**Tabela 1:** Escores obtidos por meio da avaliação dos sinais de envelhecimento facial para os grupos terapia (GT) e *biofeedback* eletromiográfico (GBE), nos diferentes momentos

	**Mínimo**		**Máximo**		**Mediana**		**Média**		**DP**	
	**GT**	**GBE**	**GT**	**GBE**	**GT**	**GBE**	**GT**	**GBE**	**GT**	**GBE**
T1	14	14	28	33	22	22	20,80	21,87	4,60	5,01
T2	10	11	36	33	17	19	18,33	20,33	6,58	6,13
T3	15	12	36	32	23	20	23,87	20,93	6,09	5,36
T4	15	11	38	31	23	21	23,20	21,20	6,18	5,39

**Legenda:GT=** grupo terapia; **GBE=** grupo *biofeedback* eletromiográfico; **T1=** 1ª avaliação; **T2=** 2ª avaliação; **T3=** 3ª avaliação; **T4=** 4ª avaliação; **mínimo=** menor escore pontuado na Análise dos Sinais do Envelhecimento Facial (ASEF); **máximo=** maior escore pontuado na ASEF; **mediana** a partir dos valores registrados na ASEF; **média e desvio padrão (DP)** a partir dos valores registrados na ASEF para a variável sinais de envelhecimento facial. **Fonte:** Elaborada pela autora.

Observou-se, na avaliação inicial T1, um grau semelhante de envelhecimento facial entre os grupos GT e GBE, registrados nos escores mínimo, máximo e mediana. Constatou-se valores numericamente distintos entre T1 e T2, com melhoria maior no GT e menor no GBE. Os escores totais mais elevados na terceira e quarta avaliações, T3 e T4, indicaram que a interrupção da realização dos exercícios e orientações, durante *washout*, resultou em aumento dos sinais do envelhecimento facial em ambos os grupos.

A análise de variância de medidas repetidas a dois critérios revelou que, em relação aos sinais de envelhecimento facial, não houve diferença estatisticamente significativa no fator grupo (p=0,81), isto é, a pontuação do GT e GBE foi semelhante, independentemente do treinamento realizado com *biofeedback* eletromiográfico. Houve, porém, uma diferença estatisticamente significativa no fator tempo (p<0,001) e na interação entre os fatores tempo e grupo (p<0,001) e o teste *Tukey* foi utilizado (Quadro 1).

**Quadro 1:** Análise da variância de medidas repetidas (ANOVA) para os sinais de envelhecimento facial

	SS	Degr. of	MS	F	p
Intercept	54528,03	1	54528,03	468,5641	0,000000
GRUPO	6,53	1	6,53	0,0561	0,814427
Error	3258,43	28	116,37		
**MOMENTO**	**176,63**	**3**	**58,88**	**12,8851**	**0,000001**
**MOMENTO/GRUPO**	**126,53**	**3**	**42,18**	**9,2304**	**0,000024**
Error	383,83	84	4,57		

**Legenda:SS:** soma dos quadrados (*sums of squares*); **degr of**: graus de liberdade (*degrees of freedom*); **MS**: variância média (*mean square*); **F:** estatística F (*F-statistic*); **p:** valor de p. **Fonte:** elaborada pela autora

Como demonstrado na Tabela 2, o teste *Tukey* indicou que, para o GBE, não foi encontrada diferença estatisticamente significativa entre valores atribuídos aos sinais de envelhecimento (letras a,b,c em T1, T2, T3 e T4), nos quatro tempos de avaliação T1 (21,87), T2 (20,33), T3 (20,93) e T4 (21,20), ou seja, as participantes não apresentaram mudança estatisticamente significativa com a intervenção proposta. Em relação ao grupo GT, o valor registrado na primeira avaliação T1 (20,80, letra a) foi estatisticamente maior que o apresentado na segunda avaliação T2 (18,33, letra c), indicativo de diminuição dos sinais de envelhecimento, após realização das nove sessões iniciais. O valor verificado na terceira avaliação T3 (23,87, letra b) foi significativamente maior que os valores das avaliações em T1 e T2, ou seja, houve aumento dos sinais de envelhecimento facial, após o período de *washout.* Não foi constatada diferença estatisticamente significativa entre os valores da quarta avaliação T4 (23,20, letras a,b) e os da primeira e terceira avaliações, indicativo de que não houve melhoria, em relação a este aspecto, após as seis sessões realizadas mensalmente.

**Tabela 2:** Análise da interação dos fatores tempo e grupo em relação aos sinais de envelhecimento facial – inter e intragrupos

**Grupo**	**Tempo**	**Média**	**DP**	**Significância** *
GBE	T1	21,87	5,01	a,b,c
GBE	T2	20,33	6,13	a,b,c
GBE	T3	20,93	5,36	a,b,c
GBE	T4	21,20	5,39	a,b,c
GT	T1	20,80	4,60	a
GT	T2	18,33	6,58	c
GT	T3	23,87	6,09	b
GT	T4	23,20	6,18	a,b

Teste Tukey (p<0,05)

* **Significância**: *letras diferentes indicam diferença estatisticamente significante*

**Legenda:GBE=** grupo *biofeedback* eletromiográfico; **GT=** grupo terapia; **T1=** 1ª avaliação; **T2=** 2ª avaliação; **T3=** 3ª avaliação; **T4=** 4ª avaliação; Média para a variável sinais de envelhecimento facial; **DP**=desvio padrão. **Fonte:** Elaborada pela autora

Na Figura 3, pode-se visualizar as mudanças que ocorreram em cada uma das avaliações realizadas.

**Figura 3:** Avaliação 1: presença de rugas glabelares, periorbitárias, acentuado sulco nasolabial, moderado sulco labiomentual; avaliação; 2: estes sinais foram atenuados; avaliação; 3: piora em rugas glabelares e periorbitárias, discreta piora em sulcos nasolabial e labiomentoniano; avaliação; 4: melhoria discreta em região dos olhos e sulco nasolabial esquerdo

Na Tabela 3 estão apresentadas as medidas descritivas, relacionadas aos escores obtidos nas avaliações das funções orofaciais (mastigação, deglutição e fala), nos distintos momentos.

**Tabela 3:** Escores obtidos por meio da avaliação das funções orofaciais (mastigação, deglutição e fala) para os grupos terapia (GT) e *biofeedback* eletromiográfico (GBE), nos diferentes momentos

	**Mínimo**		**Máximo**		**Mediana**		**Média**		**DP**	
	**GT**	**GBE**	**GT**	**GBE**	**GT**	**GBE**	**GT**	**GBE**	**GT**	**GBE**
T1	5	0	16	13	14	8	11,60	8,00	3,89	3,21
T2	2	0	14	12	7	4	6,67	4,27	3,42	3,28
T3	0	0	13	11	3	4	3,93	4,73	3,37	2,81
T4	0	0	8	12	3	5	3,27	4,67	2,05	2,97

**Legenda:GT=** grupo terapia; **GBE=** grupo *biofeedback* eletromiográfico; **T1=** 1ª avaliação; **T2=** 2ª avaliação; **T3=** 3ª avaliação; **T4=** 4ª avaliação; **mínimo=** menor escore pontuado no Protocolo MBGR; **máximo=** maior escore pontuado no Protocolo MBGR; **mediana** dos escores pontuados no Protocolo MBGR; **média e desvio padrão (DP)** dos valores registrados no Protocolo MBGR para a variável funções orofaciais. **Fonte:** Elaborada pela autora

Os escores máximos, dos dois grupos em T1, indicaram que as participantes apresentaram leves alterações nas funções orofaciais, tendo-se em vista que o máximo escore possível do Protocolo MBGR para análise deste aspecto foi 66. Observou-se em T1, que o GT apresentou escores mais elevados, ou seja, pior desempenho na avaliação inicial das funções orofaciais. Os escores obtidos nas avaliações posteriores, T2, T3, T4, indicaram que os dois grupos se beneficiaram do programa proposto. Constatou-se diminuição dos escores em T2 e estabilidade da pontuação até a conclusão do programa.

Em relação às funções orofaciais, a análise de variância de medidas repetidas a dois critérios revelou não haver diferença estatisticamente significativa entre os grupos GT e GBE (p=0,27), isto é, a pontuação destas funções foi semelhante, independentemente do treino com *biofeedback* eletromiográfico. Constatou-se uma diferença estatisticamente significativa relacionada ao fator tempo (p<0,001) e na interação dos fatores tempo e grupo (p<0,001) e o teste *Tukey* foi utilizado (Quadro 2).

**Quadro 2:** Análise da variância de medidas repetidas (ANOVA) para as funções orofaciais

	SS	Degr. of	MS	F	p
Intercept	4165,408	1	4165,408	193,4935	0,000000
GRUPO	27,075	1	27,075	1,2577	0,27161
Error	602,767	28	21,527		
MOMENTO	647,692	3	215,897	34,9272	0,00000
**MOMENTO/ GRUPO**	**132,825**	**3**	**44,275**	**7,1627**	**0,00024**
Error	519,233	84	6,181		

**Legenda: SS:** soma dos quadrados (*sums of squares*); **degr of**: graus de liberdade (*degrees of freedom*); **MS**: variância média (*mean square*); **F:** estatística F (*F-statistic*); **p:** valor de p. **Fonte:** elaborada pela autora

Como pode ser constatado na Tabela 4, por meio do teste *Tukey,* os dois grupos apresentaram uma queda significativa do valor referente às alterações das funções orofaciais, entre a primeira e segunda avaliação (em GBE, letras c,d em T1, letras a,b em T2; em GT letras d em T1 e letras b,c em T2), indicativo de melhoria nas funções orofaciais, após nove sessões iniciais, condição que se manteve estável nas terceira e quarta avaliações (em GBE, letras a,b em T3, T4; em GT letras a,b em T3, letra a em T4, revelando que não houve diferenças significativas entre estes tempos), após período de *washout* e realização de seis sessões uma vez ao mês, respectivamente. Porém, para o GT foi constatada uma diferença estatisticamente significativa entre a segunda T2 (6,67) e a quarta T4 (3,27) avaliações (letras b,c em T2, letra a em T4), com queda do valor em T4, sugerindo um resultado positivo após as seis sessões realizadas mensalmente.

**Tabela 4:** Análise da interação dos fatores tempo e grupo em relação às funções orofaciais – inter e intragrupos

**Grupo**	**Tempo**	**Média**	**DP**	**Significância** *
GBE	T1	8,00	3,21	c,d
GBE	T2	4,27	3,28	a,b
GBE	T3	4,73	2,81	a,b
GBE	T4	4, 67	2,97	a,b
GT	T1	11,60	3,89	d
GT	T2	6,67	3,42	b,c
GT	T3	3,93	3,37	a,b
GT	T4	3,27	2,05	a

Teste Tukey (p<0,05)

* **Significância** *: letras diferentes indicam diferença estatisticamente significante*

**Legenda:GBE=** grupo *biofeedback* eletromiográfico; **GT=** grupo terapia; **T1=** 1ª avaliação; **T2=** 2ª avaliação; **T3=** 3ª avaliação; **T4=** 4ª avaliação; M**édia** para a variável sinais de envelhecimento facial; **DP**=desvio padrão. **Fonte:** Elaborada pela autora.

O Questionário de Satisfação, com pontuação máxima de 90, foi respondido pelas participantes, após as nove sessões iniciais do programa proposto, antes de visualizarem suas documentações fotográficas e em vídeo, registradas nas avaliações T1 e T2. Uma vez que não houve distribuição normal dos dados, os valores registrados neste questionário foram analisados por meio do teste *Mann-Whitney* (Tabela 5).

**Tabela 5:** Análise do grau de satisfação das participantes em relação ao programa terapêutico proposto – inter-grupo

	**n**	**Perda**	**Q1**	**Mediana**	**Q3**
GBE	15	0	82,50	90,00	90,00
GT	15	0	73,50	76,00	80,00

Teste *Mann-Whitney* p<0,001.

**Legenda:GBE=** grupo *biofeedback* eletromiográfico; **GT=** grupo terapia; **n=** número de participantes; **perda=** número de desistências; **Q1=** primeiro quartil; **Q3=** terceiro quartil. **Fonte:** Elaborada pela autora

Constatou-se elevado grau de satisfação em ambos os grupos. Houve diferença estatisticamente significativa nos valores das medianas dos dois grupos (p<0,001), sendo que o grau de satisfação do GBE foi maior que o observado no GT; pode-se constatar o efeito positivo da realização do treinamento com *biofeedback* eletromiográfico neste aspecto. Verificou-se que a utilização do *biofeedback* eletromiográfico foi percebido, pelas participantes do grupo, como um recurso eficiente, complementar ao treino miofuncional orofacial. No Quadro 3, estão descritos alguns relatos sobre o efeito positivo do *biofeedback* eletromiográfico.

**Quadro 3:** Comentários das participantes do grupo GBE sobre o treinamento com o *biofeedback* eletromiográfico

**Participante**	**Comentários**
1	“Ótimo treinamento. Aprendi a comer e beber de forma que a língua trabalhe de forma correta”
2	“Essa técnica foi fundamental. A percepção sem o computador é subjetiva. A partir do momento que vejo o traçado (na tela) fica mais fácil corrigir movimentos inadequados”
3	“O *biofeedback* serviu como parâmetro para lembrar e reviver os movimentos que produzem saúde estético-bucal”
4	“Ajudou muitíssimo. Dá parâmetros se estou ativando ou não o que era necessário”
5	“Auxilia enormemente na conscientização do movimento e ritmo”
6	“Ajuda na comprovação que os exercícios estão sendo feitos corretamente”
7	“À parte o desconforto dos sensores, visualizar na tela a resposta ao movimento em tempo real foi espetacular. A compreensão dos músculos envolvidos e resposta vinculada foi impactante”
8	“Importante reeducar o cérebro para novas formas de usar as expressões faciais, movimentos doa músculos faciais. Deu uma nova autoconsciência sobre atos automáticos, que fazemos de forma errônea e não sabemos”
9	“Excelente método, pois, facilitou demais a automatização em relação à mastigação, percepção de lábios e língua no momento da deglutição”
10	“O treinamento me ajudou a fixar os movimentos da língua e mordida. Quando visualizei esses movimentos achei mais fácil quando fazia em casa”
11	“A memória visual potencializa a repetição dos exercícios em casa”
12	“Este treinamento ajuda a enxergarmos o momento da mastigação e deglutição e como pode ser melhorado”
13	“Muito interessante. Tive dificuldade em realizar alguns movimentos e também de controlar os músculos da face, mas acredito que com o tempo vou superar todas as dificuldades”
14	“O *biofeedback* ajuda muito na dissociação dos movimentos e também no retorno da execução”
15	“O treinamento me ajudou a perceber como exatamente o exercício/ movimento deve ser feito, fica registrado na minha memória para o uso na rotina de alimentação e movimentos”

**Legenda: GT=** grupo terapia; **GBE=** grupo *biofeedback* eletromiográfico.

**Fonte:** Elaborada pela autora

## DISCUSSÃO

O número de fonoaudiólogos que atuam no campo da estética facial tem se ampliado nos últimos anos, porém, não foram encontradas publicações de ensaio clínico randomizado sobre este tema, na Fonoaudiologia. Os estudos neste campo de atuação são escassos e, em sua maioria, apresentaram descrições com número reduzido de sujeitos e falhas metodológicas, que inviabilizam a reprodução dos procedimentos propostos^(10)^.

Os ensaios clínicos randomizados, considerados padrão de referência como método para a investigação e comprovação da eficácia, são raros na área de Motricidade Orofacial. A presente pesquisa representa o primeiro ensaio clínico randomizado em que o *biofeedback* eletromiográfico foi utilizado, no campo da Fonoaudiologia em estética facial. Na presente pesquisa, não houve desistência de participantes, podendo-se justificar a positiva adesão pelo rigoroso processo de seleção das participantes, efetivo esclarecimento sobre procedimentos metodológicos envolvidos e regras claras sobre a necessidade de execução diária das orientações prescritas em terapia, assim como da permanência nas diferentes etapas da pesquisa.

Os testes estatísticos revelaram que a maioria das participantes da presente pesquisa apresentaram alterações leves em relação aos sinais de envelhecimento facial avaliados, diferentemente do esperado na faixa etária pesquisada – 50 a 60 anos de idade. Provavelmente, o estilo de vida saudável, predominante no grupo das voluntárias, assim como suas preferências por adoção de cuidados estéticos naturais, justificaram estes resultados.

O escore total aferido revelou que apenas para o grupo GT houve mudança significativa em relação aos sinais de envelhecimento facial. Neste grupo os escores registrados na segunda, terceira e quarta avaliações revelaram, respectivamente, melhoria dos sinais de envelhecimento facial após realização das nove sessões iniciais, piora com a interrupção de oito semanas, resultado que se manteve praticamente inalterado após seis sessões realizadas mensalmente, havendo apenas melhoria da ptose mandibular e das rugas frontais estáticas. Para o grupo GBE, no período final de seis meses, houve melhoria na função sorriso, com consequente atenuação das rugas periorbitárias dinâmicas. Porém, essas melhorias estéticas foram consideradas reduzidas tendo-se em vista os inúmeros aspectos contemplados no tratamento.

Uma vez que não foi constatada piora nos padrões de mastigação e deglutição aprendidos pelas participantes de ambos os grupos, o resultado de pontuação com maior prejuízo quanto aos aspectos estéticos, no período de seis meses após o *washout*, poderia estar relacionado tanto à interrupção no uso da bandagem inelástica Transpore^TM^, dos exercitadores facial e lingual, do garrote, do exercício “fala em rolha”, quanto ao provável desânimo das participantes na segunda etapa de intervenção, com a constatação das perdas estéticas apresentadas após a terceira avaliação.

A relação entre sinais de envelhecimento facial e contração excessiva dos músculos faciais foi descrita por vários profissionais. Para alguns fonoaudiólogos, haveria diminuição dos sinais de envelhecimento facial com adequação das funções orofaciais^(5-7)^. A análise do desempenho das participantes em relação às mudanças ocorridas nas funções orofaciais avaliadas, comprovada pelos testes estatísticos aplicados, permitiu interrogar esta relação. No presente trabalho, constatou-se melhoria e estabilidade dos padrões de mastigação e deglutição aprendidos, até a conclusão da pesquisa; pode-se afirmar que houve automatização destes padrões, sem haver mudança correspondente nos sinais de envelhecimento facial, cujos escores aumentaram após *washout*, ou seja, em nenhum dos aspectos estéticos avaliados observou-se relação com a melhoria nas funções orofaciais.

Nas publicações consultadas esta correlação foi relatada de forma genérica, isto é, sem referência à detecção de um aspecto alterado na avaliação, que tenha sido corrigido com uma intervenção específica^(5-7)^. Assim, pode-se afirmar que, os escores dos sinais de envelhecimento facial, indicando melhoria na segunda avaliação (principalmente rugas frontais estáticas) e acentuação dos sinais em T3 (rugas periorbitárias, periorais estáticas, ptose submandibular e sulco labiomentoniano), referem-se ao efeito de procedimentos específicos realizados na primeira etapa do programa proposto e à interrupção destes no período de *washout,* quando foram recolhidos todos os materiais utilizados em terapia e, também, a folha contendo as orientações prescritas para execução dos exercícios e treinamento funcional em domicílio. Neste aspecto, os procedimentos utilizados mostraram-se eficientes para atenuar rugas e sinais de envelhecimento no terço superior da face. A intervenção fonoaudiológica mostrou-se uma alternativa para atenuar os sinais de envelhecimento facial, além dos procedimentos dermatológicos já existentes para diminuir a contração excessiva dos músculos mímicos^(9)^.

Na 10ª semana da presente pesquisa, as participantes responderam ao Questionário de Satisfação, que continha questões relativas ao programa terapêutico, tais como: grau de satisfação com resultados obtidos, expectativas, metas atingidas, desempenho da terapeuta e possibilidade de indicação deste programa para outros indivíduos. Os resultados estatísticos revelaram que as participantes de ambos os grupos ficaram satisfeitas com os resultados do programa aplicado, sendo maior o grau de satisfação do GBE.

A avaliação da satisfação das participantes em relação ao programa foi um procedimento importante, tendo-se em vista que a insatisfação com a aparência é o fator que guia o cliente na procura por uma intervenção estética facial^(26)^. Os tratamentos estéticos têm impacto positivo na saúde emocional dos indivíduos. Muitos buscam tratamentos minimamente invasivos, para atenuar o envelhecimento facial, que os deixem com a aparência natural e relaxada^(26)^.

Diferentemente do presente estudo, em que as participantes responderam ao questionário antes de compararem as documentações fotográficas e em vídeos realizadas na primeira e segunda avaliações, na maioria das publicações em que a satisfação dos indivíduos foi investigada, a autoavaliação foi realizada por meio da comparação entre as imagens fotográficas realizadas antes e depois da realização dos procedimentos estéticos^(26-28)^.

Os resultados aferidos no protocolo MBGR e na ASEF revelaram a persistência de alterações funcionais e de sinais de envelhecimento facial, mesmo após as nove semanas iniciais de intervenção. No entanto, o Questionário de Satisfação demonstrou que houve resposta positiva das participantes em relação ao tratamento miofuncional orofacial e estético recebido. Esta divergência entre os resultados observados após a intervenção e o nível de satisfação das participantes foi igualmente destacada em uma pesquisa conduzida por cirurgiões plásticos^(27)^.

No presente estudo, constatou-se alto grau de satisfação em relação aos procedimentos não invasivos utilizados, que promoveram leves mudanças estéticas e mudanças funcionais mais acentuadas. Este resultado corrobora observações de autores, para quem mudanças sutis na aparência podem afetar substancialmente a autoestima e felicidade dos indivíduos^(28)^.

A presente pesquisa representa o primeiro ensaio clínico randomizado no campo da Fonoaudiologia em estética facial em que o *biofeedback* eletromiográfico foi utilizado. Alguns pesquisadores afirmaram que uma das vantagens do *biofeedback* eletromiográfico seria a visualização da atividade muscular, representada na tela do computador, possibilitando que os pacientes tivessem participação mais ativa com maior controle sobre a musculatura orofacial^(15,16)^. No presente estudo, as respostas registradas no Questionário de Satisfação constataram esta vantagem: as integrantes do GBE puderam aprender a controlar o recrutamento dos músculos trabalhados durante o treinamento da mastigação, deglutição e sorriso. Vale lembrar que eletrodos posicionados na região do risório, para o treinamento do sorriso, não impedem a captação do sinal elétrico dos músculos adjacentes (*cross talk*) zigomáticos maior, menor e bucinador. Ainda assim, as participantes conseguiam comparar a contração dos músculos desta região e da porção orbital inferior do músculo orbicular do olho e, por meio de controle voluntário e visualização do sinal na tela do computador, diminuir a contração muscular na região dos olhos ao sorrir, com consequente atenuação de rugas periorbitárias estáticas e dinâmicas.

Os efeitos positivos deste recurso para reduzir contração dos músculos envolvidos na mastigação, deglutição e articulação da fala e consequentemente, diminuir as rugas faciais foram descritos, até o momento, em uma única publicação sobre uso do *biofeedback* eletromiográfico na terapia fonoaudiológica em estética facial^(14)^.

Uma hipótese formulada no presente estudo foi que os participantes do GBE, obteriam escores superiores aos do GT. Porém, não houve diferença significativa na comparação do desempenho entre os dois grupos. Ambos apresentaram melhoria e manutenção dos padrões de mastigação e deglutição aprendidos, sem modificação com relação ao sorriso e fala.

Em relação aos sinais de envelhecimento facial, a pontuação do GT e GBE foi semelhante nos diversos momentos avaliados, independentemente do treinamento realizado com *biofeedback* eletromiográfico.

Constatou-se diferença entre os grupos no grau de satisfação em relação aos resultados obtidos após a realização da primeira etapa do programa, sendo que no GBE o grau de satisfação foi maior. As participantes relataram que a visualização na tela do computador, da imagem correspondente à atividade muscular treinada, favoreceu a conscientização, o controle e o aprendizado dos movimentos necessários para a realização dos padrões adequados de mastigação, deglutição e mímica facial durante as sessões e a fixação destes padrões em suas residências. O impacto positivo proporcionado pelo *feedback* visual foi, também, relatado por sujeitos saudáveis e disfágicos em outro estudo, na área da disfagia^(16)^.

Apesar da concordância que o treinamento com *biofeedback* eletromiográfico potencializou a contração muscular de sujeitos com algumas alterações miofuncionais orofaciais, não foram encontrados, até o momento, estudos comprovando diferenças entre desfechos finais de intervenções realizadas com e sem a associação deste recurso terapêutico^(15)^.

O *washout*, recurso pouco utilizado em pesquisas fonoaudiológicas^(^^29^^)^, foi importante na presente pesquisa para verificação das perdas após interrupção do programa terapêutico. Uma das hipóteses formuladas no presente estudo foi que haveria perdas funcionais e estéticas com a interrupção dos exercícios, hipótese não confirmada em relação às funções mastigação e deglutição, uma vez que os padrões aprendidos na primeira etapa do programa proposto foram integrados, nem em relação às funções fala e sorriso, cujos escores mantiveram-se praticamente sem alteração do início ao final da pesquisa. Entretanto, constatou-se perda estética e acentuação das rugas periorais e periorbitárias estáticas, ptose mandibular, para as participantes do GT e acentuação do sulco labiomentoniano e rugas frontais estáticas, em ambos os grupos, confirmando que as participantes não realizaram os exercícios durante esse período. A interrupção dos exercícios propostos foi, provavelmente, a causa destas perdas estéticas. Uma vez que o envelhecimento é inexorável, sugere-se que a execução rotineira de exercícios fonoaudiológicos específicos pode ser benéfica para atenuar alguns sinais de envelhecimento facial.

Os resultados do presente estudo sugerem que, em oposição ao apontado pelos estudos publicados até o presente momento^(6,7)^, ainda que inadequada, a contração intermitente dos músculos periorais durante as funções de mastigação, deglutição e fala não resultaria necessariamente em maior incidência de rugas nesta região. Pode-se concordar parcialmente com estes estudos, quanto à correlação entre diminuição da contração exagerada dos músculos mímicos e redução das rugas faciais. Esta associação foi encontrada, no presente estudo, especificamente em relação às rugas frontais estáticas. Considera-se, entretanto, a adequação das funções orofaciais um fator importante para promoção do envelhecimento saudável, independentemente do impacto destas sobre a estética facial.

A proposta de realização de uma segunda etapa da pesquisa foi elaborada a partir da hipótese formulada de que a interrupção dos exercícios no período do *washout* acarretaria perdas estéticas e funcionais, que seriam superadas com as seis sessões realizadas mensalmente. Não foram encontradas pesquisas, na área da Fonoaudiologia, que apresentassem proposta semelhante.

Em relação às funções orofaciais, as alterações observadas na avaliação inicial foram praticamente superadas na primeira fase do programa, não sendo constatado piora dos padrões de mastigação e deglutição aprendidos, após o período de *washout*, em ambos os grupos.

Em relação aos sinais de envelhecimento facial, observou-se queda dos escores em alguns aspectos, que indicaram efeitos positivos desta proposta, após as seis sessões realizadas mensalmente. Para o GT houve melhoria da ptose mandibular e das rugas frontais estáticas, enquanto para o grupo GBE houve melhoria na função sorriso, ou seja, nas rugas periorbitárias dinâmicas. Tendo-se em vista o aumento dos escores em quase todos os aspectos avaliados após período de *washout*, principalmente no grupo GT, foram reduzidas as mudanças estéticas observadas na segunda etapa do programa. Considerando-se que o envelhecimento facial é progressivo, sugere-se que os procedimentos fonoaudiológicos para atenuar sinais de envelhecimento sejam mantidos sem interrupção.

A presente pesquisa trouxe contribuições importantes para a Fonoaudiologia, tanto na área de Motricidade Orofacial, quanto no campo de atuação em estética facial. Constatou-se que o treino funcional para adequar as funções orofaciais de mastigação e deglutição, realizado em nove sessões, foi suficiente para que os padrões motores aprendidos fossem integrados/automatizados, não havendo perdas funcionais após o período de washout; o programa testado mostrou-se eficaz para atenuar as rugas e sinais de envelhecimento facial; os resultados apresentados mostraram as possibilidades e limitações deste programa e, desta forma, apontam caminhos para futuras pesquisas no campo da Fonoaudiologia em estética facial, em que são escassas as publicações científicas.

### Limitações do estudo

Conforme citado anteriormente, a maioria das participantes da pesquisa apresentaram alterações leves em relação aos sinais de envelhecimento facial avaliados, sendo assim, novos estudos poderão aplicar o programa em mulheres acima de 60 anos de idade. Sugere-se, também, em futuras pesquisas, a inclusão de grupo controle e que se evite apresentação dos resultados parciais às participantes (comparação da primeira avaliação com a segunda) antes da realização da terceira e quarta avaliações, prevenindo interferência na avaliação das participantes sobre o tratamento realizado, a partir das informações obtidas.

A intervenção proposta no presente estudo contemplou apenas uma das estruturas que se modificam com o envelhecimento, ou seja, os músculos faciais. Acredita-se, que resultados mais positivos poderiam ser alcançados associando-se a terapia fonoaudiológica a outros tratamentos, como os tratamentos propostos por dermatologistas, cujos procedimentos, mais ou menos invasivos, incluem intervenções para reparar as mudanças estruturais inerentes ao envelhecimento facial.

## CONCLUSÃO

O programa proposto resultou em mudanças estéticas, atenuação dos sinais de envelhecimento, principalmente, para as participantes do grupo GT e mudanças funcionais na mímica facial, adequação da mastigação e deglutição, sem alteração nas funções fala e sorriso, em todas as participantes da pesquisa (GT e GBE), após nove sessões realizadas semanalmente. A interrupção do programa por oito semanas resultou em perdas estéticas, mas não em perdas funcionais, para os grupos GT e GBE. O uso do *biofeedback* eletromiográfico resultou em escores superiores para o GBE, comparado ao GT, apenas em relação ao grau de satisfação, não tendo impactado no treinamento dos padrões de mastigação, deglutição e sorriso. O grau de satisfação das participantes foi superior aos resultados aferidos no Protocolo MBGR e na ASEF. As seis sessões realizadas mensalmente, após o período de *washout,* tiveram efeito limitado na superação das perdas estéticas.
